# LncRNA SPANXA2-OT1 Participates in the Occurrence and Development of EMT in Calcium Oxalate Crystal-Induced Kidney Injury by Adsorbing miR-204 and Up-Regulating Smad5

**DOI:** 10.3389/fmed.2021.719980

**Published:** 2021-09-27

**Authors:** Haiyan Hu, Jie Zhang, Yinhui Li, Jiarong Ding, Wei Chen, Zhiyong Guo

**Affiliations:** ^1^Department of Nephrology, Changhai Hospital, The Naval Military Medical University, Shanghai, China; ^2^Department of Nephrology, Hainan Hospital of Chinese PLA General Hospital, The Hainan Academician Team Innovation Center, Sanya, China

**Keywords:** crystalline kidney injury, epithelial-mesenchymal trans-differentiation, lncRNA SPANXA2-OT1, miR-204, Smad5

## Abstract

**Objective:** To explore the regulatory mechanism of long non-coding RNAs (lncRNAs) in the occurrence and development of epithelial-mesenchymal transition (EMT) in calcium oxalate crystal-induced kidney injury.

**Materials and Methods:** Gene core technique was used to screen differentially expressed lncRNAs and mRNAs in HK-2 cells before and after calcium oxalate monohydrate (COM) stimulation; differentially expressed mRNAs were then analyzed using GO and pathway analysis. The role of target lncRNA in EMT in renal tubular epithelial cells induced by COM was further investigated by applying a series of *in vitro* experiments.

**Results:** Four differentially expressed lncRNAs (ABCA9-AS1, SPANXA2-OT1, RP11-955H22.1, and RP11-748C4.1) were up-regulated after 48 h of COM stimulation compared to the control group, where up-regulated expression of lncRNA SPANXA2-OT1 was the most significant. Thus, lncRNA SPANXA2-OT1 was further examined. Interference lncRNA SPANXA2-OT1 reversed the down-regulation of E-cadherin and Pan-ck, and up-regulated Vimentin and α-SMA induced by COM stimulation. The application of miR204 inhibitor weakened the interference effect of interfering RNA on lncRNA SPANXA2-OT1 and promoted the occurrence of EMT. Moreover, the miR204 simulator alleviated the overexpression effect of lncRNA SPANXA2-OT1 on COM-stimulated renal tubular epithelial cells and inhibited the occurrence of EMT in renal tubular epithelial cells. Also, a dual-luciferase reporter assay showed that miR-204 could bind to lncRNA SPANXA2-OT1 and Smad5, while lncRNA SPANXA2-OT1 could inhibit cell proliferation and promote cell apoptosis.

**Conclusion:** The lncRNA SPANXA2-OT1 is involved in the occurrence and development of EMT in renal tubular epithelial cells induced by crystalline kidney injury by adsorbing miR-204 and up-regulating Smad5.

## Introduction

The formation of renal stones and the exact mechanism related to the renal stone injury are still unclear. Previous studies have shown that epithelial-mesenchymal transition (EMT) is an important initiation link of renal interstitial fibrosis (RIF) and suggested that EMT has an important role in renal tissue damage repair (1, 2). Our previous studies also indicated that EMT occurs in renal tubular epithelial cells at the initial stage of renal stone formation or the stage of crystallize-induced renal injury, triggering the process of renal fibrosis (3, 4). However, the molecular mechanism of EMT triggered by crystalline renal injury is still not fully understood.

Long non-coding RNA (lncRNAs) are a type of non-coding RNAs that can regulate gene expression (5–8) and have important roles in various cancers. Recent studies have suggested an association between lncRNA and tumor invasion and migration (type III EMT) (9–11). Moreover, lncRNAs have been shown to have a critical role in the process of organ fibrosis (12, 13). We speculated that lncRNA might also have a very important regulatory role in the occurrence and development of type II EMT in the early stage of renal fibrosis. In this study, we examined the regulatory mechanism of lncRNAs in the occurrence and development of EMT in calcium oxalate crystal kidney injury.

## Materials and Methods

### Cell Culture and Treatment

Cell culture and treatment were performed as previously described (4, 14). HK-2 cells were cultured in DMEM/F12 (HyClone) containing 10% fetal bovine serum (FBS, Gibco), 100 U/ml penicillin, and 100 μg/ml streptomycin, in 5% carbon dioxide at a temperature of 37°C. Cells were then exposed to calcium oxalate monohydrate (COM; 200 μg/ml, Sigma, USA) for 48 h. Calcium oxalate monohydrate crystals were sterilized by heating overnight at 180°C as previously described (15). The used renal tubular epithelial cells (HK-2) were cryopreserved by our laboratory for early passage. Interfering RNA, overexpression virus, miRNA-inhibitor, miRNA-mimics dual fluorescein reporter gene plasmids were all purchased from Jiman Biological Co., Ltd. (Shanghai).

### Microarray Hybridization and Data Analysis

Arraystar Human LncRNA Microarray V4.0 is designed for the global profiling of human lncRNAs and protein-coding transcripts. Agilent Feature Extraction software (version 11.0.1.1) was used to analyze acquired array images. Quantile normalization and subsequent data processing were performed using the GeneSpring GX v12.1 software package (Agilent Technologies). Differentially expressed lncRNAs and mRNAs between the two samples were identified through Fold Change filtering. Pathway analysis and GO analysis were applied to determine the role of differentially expressed mRNAs in these biological pathways or GO terms. The genome expression profile chip, the chip hybridization map, and the chip data analysis were completed by Shanghai Kangcheng Biological Co., Ltd.

### Quantitative Real-Time Polymerase Chain Reaction

Real-time polymerase chain reaction (RT-PCR) was performed as previously described (14). Briefly, total RNA from HK-2 cells was extracted with trizol and then reverse-transcribed using a kit. Real-time PCR was performed using the StepOnePlus™ RT-PCR System (Applied Biosystems) with TaqMan SYBR Green. All reagents for quantitative real-time polymerase chain reaction (qRT-PCR) were obtained from TaKaRa, Bio group, and utilized according to the manufacturer's protocol. The primer sequences are shown in Supplementary Table 1.

### Immunofluorescence

Immunofluorescence was performed as previously described (16). Cells were cultured on coverslips overnight and fixed with 4% paraformaldehyde for 20 min at room temperature. Samples were then incubated with 0.3% Triton X-100 for 10 min and blocked for 2 h with the blocking solution (Beyotime), after which cells were probed overnight at 4°C with a diluted primary antibody, followed by a secondary antibody for 2 h. The primary antibodies used were: a mouse monoclonal antibody for Pan-ck (1:300, Abcam), Vimentin (1:150, Santa Cruz), and rabbit polyclonal antibody against E-cadherin (1:100, Abcam), α-SMA (1:100, Abcam), and Smad5 (1:100, Abcam); the secondary antibodies used were Alexa 488-conjugated anti-mouse IgG (Invitrogen, Carlsbad, CA) and Alexa 594-conjugated anti-rabbit IgG (Invitrogen, Carlsbad, CA).

### Western Blot

Western blot was performed as previously described (14). Total proteins were extracted from HK-2 cells using RIPA lysis buffer (Beyotime, Haimen China) and separated by 12.5% sodium dodecyl sulfate-polyacrylamide gel electrophoresis (SDS-PAGE). The protein lysates were transferred onto a 0.22-μm PVDF membrane (Millipore). After blocking in 5% non-fat milk at room temperature for 2 h, the membranes were incubated with primary antibodies specific for E-cadherin (Abcam), α-SMA (Abcam), Vimentin (Santa Cruz), Pan-ck (Abcam), Smad5 (Abcam), and GAPDH (Cell signaling) at 4°C overnight. After three washes, the blots were incubated with IRDye 700/800-conjugated secondary antibodies. The results were visualized and analyzed using an Odyssey infrared scanner.

### Dual-Luciferase Reporter Assay

HK-2 cells were seeded in a 6-well plate (1.0 × 10^6^ cells/well) and incubated for 48 h. Next, cells were transfected with pGL3-SPANXA2-OT-1 WT, pGL3-SPANXA2-OT-1 MT, pGL3-Smad5-3′UTR WT, or pGL3-Smad5-3′UTR MT vectors together with miR-204-5p or miR-NC. After 48 h, the cells were harvested and subjected to the luciferase reporter assay using the Dual-Luciferase Reporter Assay System (Promega) according to manufacturer's instructions.

### Cell Proliferation Assay (CCK-8)

Cell proliferation was assessed using a cell counting kit-8 (CCK-8, Dojindo Laboratories) according to the protocols supplied by the manufacturer. Briefly, HK-2 cells were plated in 96 well plates (8 × 10^3^ cells/well) and incubated overnight. Then, the cell medium was replaced with DMEM/F12 with or without COM (200 μg/ ml), and cells were incubated for 12, 24, and 48 h. At each time point, 10 μl of sterile CCK-8 reagent was added to each well and incubated for another 2 h at 37°C. The optical density (OD) of each well was measured at 450 nm to calculate the number of living cells using a multimode reader (BioTek). Each experiment was run in triplicate.

### TUNEL Assay

The cells cultured in 24-well plates were washed with PBS 3 times and fixed with 4% paraformaldehyde for 30 min. After washing once with PBS, samples were incubated with 0.5% Triton X-100 PBS at room temperature for 10 min. After washing with PBS three times, the TUNEL detection solution containing TdT enzyme and fluorescent labeling solution was added, and the samples were incubated at 37°C for 1 h in dark. Then, samples were washed with 0.5% Triton X-100 PBS three times and stained with DAPI at room temperature for 10 min in dark. Consequently, samples were washed with 0.5% Triton X-100 PBS three times before; the bottom of the wells was washed with distilled water. The cell sliver was then collected and fixed on the slide with anti-fluorescence quenching sealing solution. After sealing, the samples were analyzed under a fluorescence microscope.

### Statistical Analysis

SPSS17.0 statistical software was used to process the data, and GraphPad Prism 5.0 software was used to make the images. For the normal distribution, ANOVA analysis was used; for non-normal distribution, a non-parametric statistical method was applied (H-test). A *p*-value of ≤ 0.05 was considered statistically significant.

## Results

### HK-2 Cells Stimulated Using Calcium Oxalate Monohydrate

**Figure 1:**
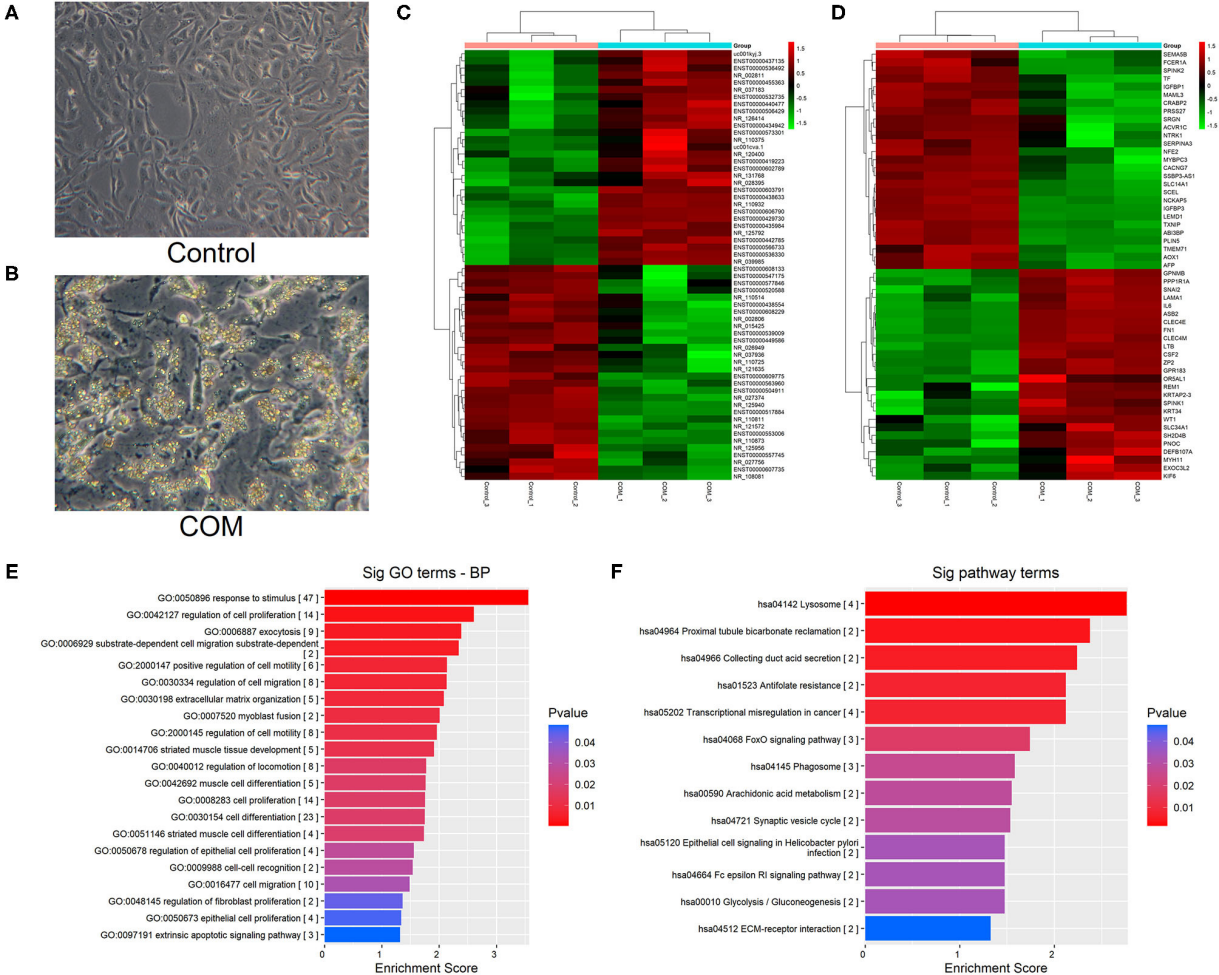
**(A,B)** Cell morphology before and after COM stimulation [control group **(A)** vs. COM group **(B)**; 400 x]. **(C)** Cluster analysis of lncRNAs. **(D)** Cluster analysis of mRNAs. **(E)** The biological function of differentially up-regulated mRNA. **(F)** The enrichment analysis of the main pathway corresponding to the up-regulated genes. A *p* < 0.05 indicates that the difference is statistically significant. The red bands represent relatively high expression levels, and the green bands represent relatively low expression levels.

In control group, the cells had full fusiform shape, while in COM group, the HK-2 cells changed from a full fusiform shape to a thin and long strip shape, there were many crystals attached to the surface of the cell. At the same time, the number of cells was significantly reduced (Figures 1A,B).

### GeneChip Analysis of HK-2 Cells Before and After Calcium Oxalate Monohydrate Stimulation

#### Cluster Analysis of lncRNA and mRNA Chip

Different lncRNAs and mRNAs were screened in HK-2 cells treated with COM stimulation after 48 h. A total of 197 differentially up-regulated and 501 differentially down-regulated lncRNAs were detected by cluster analysis heatmap. Meanwhile, 80 differentially up-regulated mRNAs and 148 differentially down-regulated mRNAs were screened by bio-enrichment analysis (Figures 1C,D).

#### Differential mRNAs Were Analyzed by GO and KEGG Pathway

GO analysis of differentially expressed mRNAs showed that HK-2 cells were more active in migration, cell proliferation regulation, cell differentiation, and intercellular recognition after COM stimulation (Figure 1E). Furthermore, the KEGG pathway enrichment analysis showed that the expression of laminin and fibronectin was significantly up-regulated in the signaling pathways related to the interaction between extracellular matrix receptors in HK-2 cells after COM stimulation (Figure 1F).

### LncRNAs Analysis of Differential Expression

According to the sources of the differentially up-regulated lncRNAs (Refseq, UCSC known genes, Gencode) and fold change ≥2, eight up-regulated lncRNAs were screened, as shown in Table 1.

**Table 1:** LncRNAs up-regulated in the COM group compared with the control group.

**Gene symbol**	**Seq name**	***p*-value**	**FDR**	**Fold change**	**Regulation**	**Type**
ABCA9-AS1	NR_126414	0.010194086	0.198432673	3.0315399	Up	Non-coding
LOC105667213	NR_131768	0.029169914	0.300753128	2.9038507	Up	Non-coding
SPANXA2-OT1	NR_037183	0.033326824	0.308559486	2.5742044	Up	Non-coding
RP11-955H22.1	ENST00000536330	0.000583373	0.089535377	2.2445738	Up	Non-coding
RP11-748C4.1	ENST00000532735	0.037944481	0.322917618	2.1281075	Up	Non-coding
AC104135.3	ENST00000435984	0.00113302	0.097207568	2.0381036	Up	Non-coding
LINC01291	NR_125792	0.001082149	0.094140318	2.0258946	Up	Non-coding
CTD-2130F23.1	ENST00000506429	0.010892477	0.202846548	2.0228507	Up	Non-coding

### Target lncRNA SPANXA2-OT1

**Figure 2:**
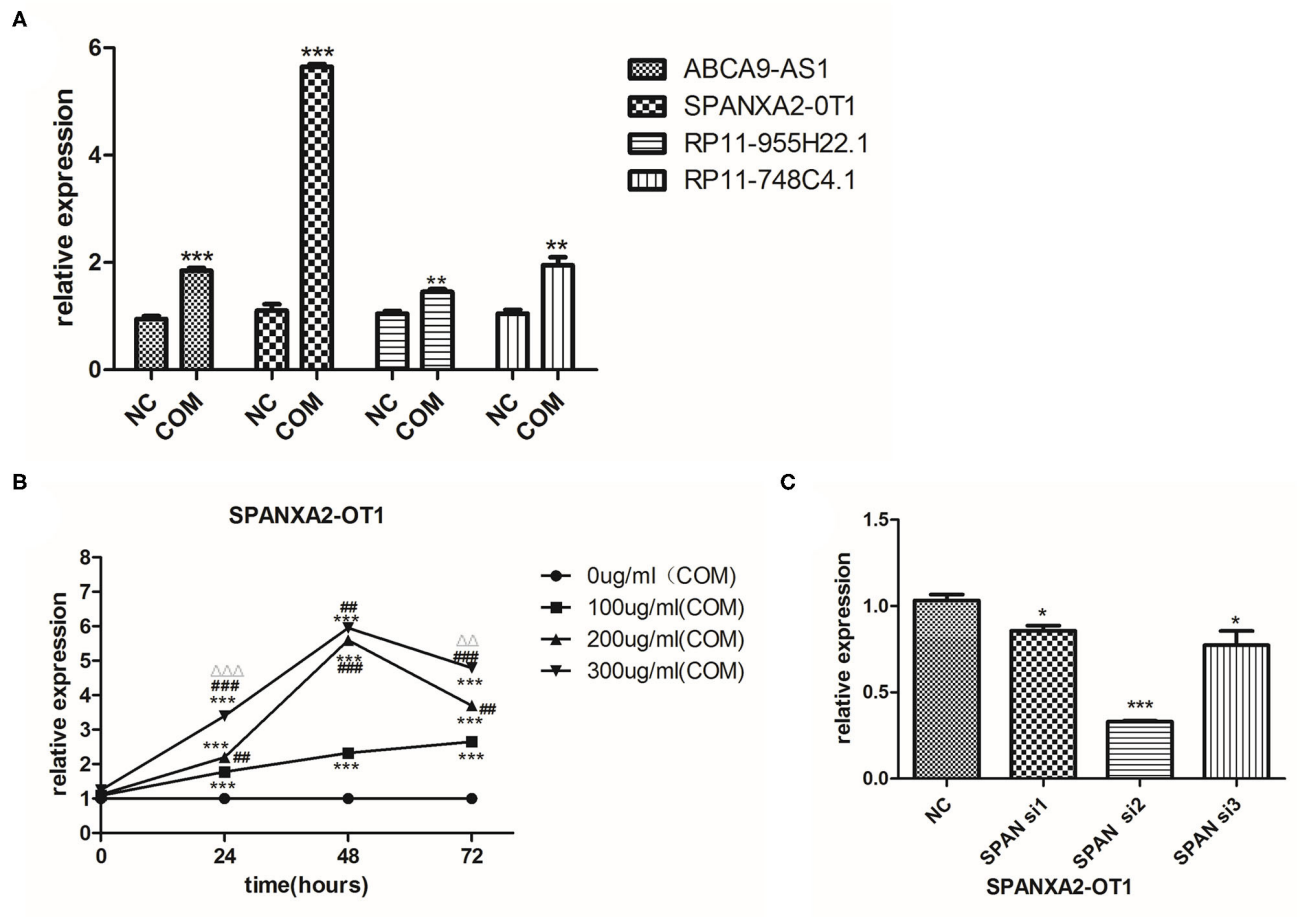
**(A)** Relative expression of lncRNAs in the two groups. NC, control group; COM, calcium oxalate monohydrate stimulated group; *p < 0.05, **p < 0.01, ***p < 0.0001. **(B)** The expression of LncRNA SPANXA2-OT1 in HK-2 cells (exposed to different COM concentrations and different stimulation times).***p < 0.001 vs. 0 μg/ml (COM) group, ^##^*p* < 0.01 vs. 100 μg/ml (COM) group, ^###^*p* < 0.001 vs. 100 μg/ml (COM) group, ^ΔΔ^*p* < 0.01 vs. 200 μg/ml (COM) group, ^ΔΔΔ^*p* < 0.001 vs. 200 μg/ml (COM). **(C)** Verification of the efficiency of interference RNA; control group (NC), interference group 1 (SPAN si1), interference group 2 (SPAN si2), and interference group3 (SPAN si3). *p < 0.05 vs. NC group, ***p < 0.0001 vs. NC group.

Four differentially expressed lncRNAs were screened by RT-PCR, namely ABCA9-AS1, SPANXA2-OT1, RP11-955H22.1, and RP11-748C4.1. The results of RT-PCR showed that the expression of these four lncRNAs was up-regulated after 48 h of COM stimulation compared to the control group, and the up-regulated expression of SPANXA2-OT1 was the most significant (*p* < 0.0001, Figure 2A). These results suggested that lncRNA SPANXA2-OT1 is responsible for the occurrence and development of EMT caused by calcium oxalate crystal kidney injury; thus, this lncRNA was taken as the target lncRNA for subsequent functional verification and preliminary mechanism exploration.

### The Expression of SPANXA2-OT1 in HK-2 Cells After Different COM Concentrations and Different Times Stimulation

The expression of SPANXA2-OT1 increased in the first 48 h after COM stimulation in a dose-dependent and time-dependent manner; yet, no statistical difference was observed in the expression of lncRNA after stimulation with 200 and 300 μg/ml COM for 48 h. As the expression of SPANXA2-OT1 started to decrease after 48 h (Figure 2B), a 48 h stimulation with a COM concentration of 200 μg/ml was selected for the next experiment.

### Using Interventional Assay to Verify the Involvement of lncRNA SPANXA2-OT1 in the Occurrence of EMT in HK-2 Cells Induced by Crystal Kidney Injury

**Figure 3:**
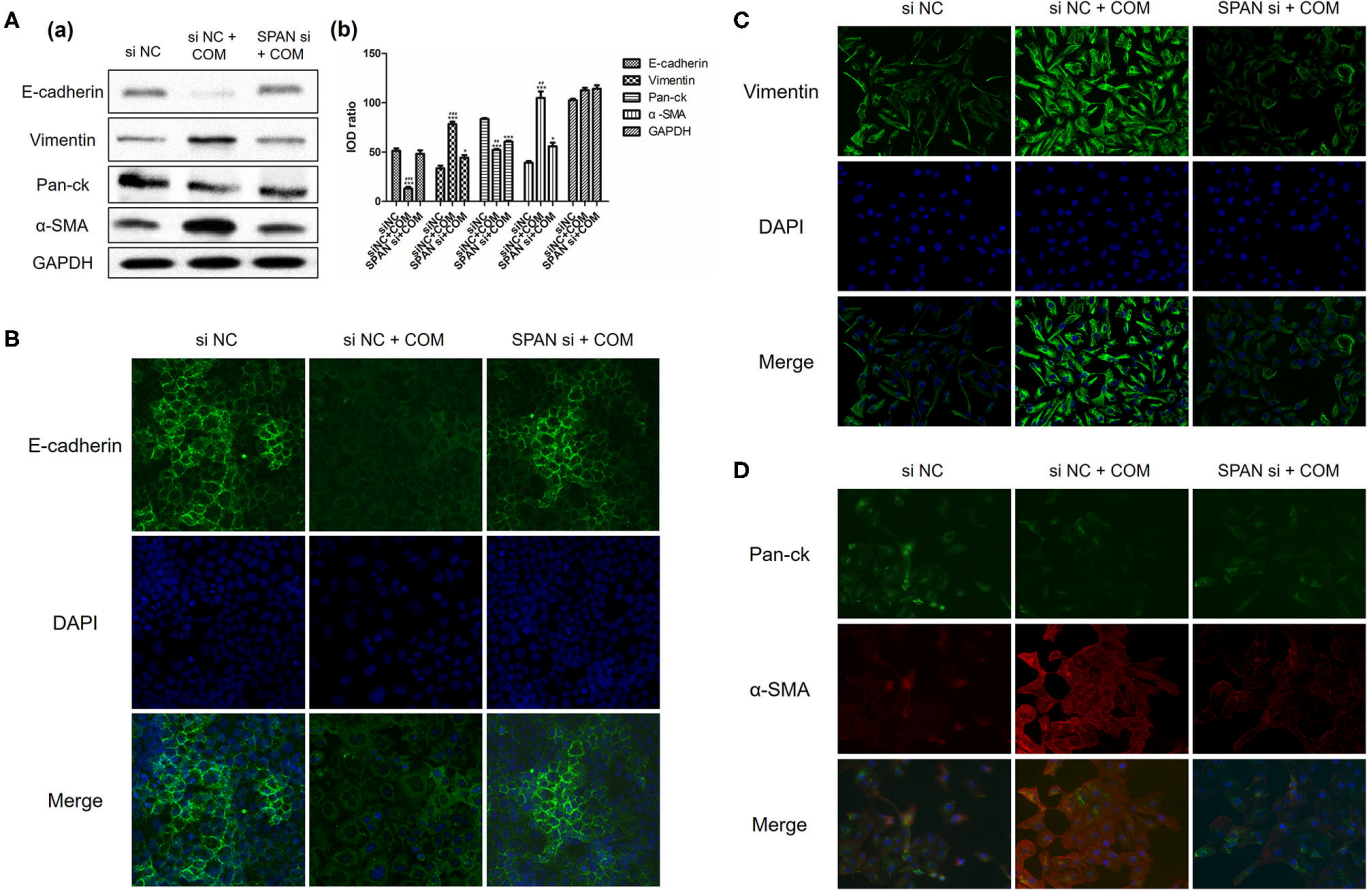
**(A)** Expression of E-cadherin, Vimentin, Pan-ck, and α-SMA in each group [(a), Western blot; (b), Western blot semi-quantitative]. siNC. negative interference control group; siNC + COM. negative interference model group; SPAN si + COM. interference model group (after small interfering RNA interferes with lncRNA SPANXA2-OT1). *p < 0.05 vs. siNC group, ***p < 0.001 vs. siNC group, ^##^*p* < 0.01 vs. SPAN si + COM group, ^###^*p* < 0.001 vs. SPAN si + COM group. **(B)** Immunofluorescence staining results of cells in each group (E-cadherin, green; DAPI, blue; 200 x). siNC, negative interference control group; siNC + COM, negative interference model group; SPAN si + COM, interference model group (after small interfering RNA interferes with lncRNA SPANXA2-OT1). **(C)** Immunofluorescence staining results of cells in the group (vimentin, green; DAPI, blue; 200 x). siNC, negative interference control group; siNC + COM, negative interference model group; SPAN si + COM, interference model group (after small interfering RNA interferes with lncRNA SPANXA2-OT1). **(D)** Immunofluorescence staining results of each group (Pan-ck, green; α-SMA, red; DAPI, blue; 200×). siNC, negative interference control group; siNC + COM, negative interference model group; SPAN si + COM, interference model group (after small interfering RNA interferes with lncRNA SPANXA2-OT1).

Three small interfering RNAs were designed, and the small interfering RNA with the highest interference efficiency of lncRNA SPANXA2-OT1 was selected by RT-PCR for subsequent experiments (Figure 2C). Western blot and immunofluorescence were applied to detect the expressions of epithelial phenotypic markers (Pan-ck and E-cadherin) and stromal markers (Vimentin and α-SMA) in each group. The results showed that the expression of epithelial marker E-cadherin and Pan-ck was decreased in the negative interference model group (siNC + COM) after calcium oxalate stimulation compared with the negative interference control group (siNC), while the expression of stromal marker Vimentin and α-SMA was up-regulated. Compared with the negative interference model group, epithelial marker E-cadherin and Pan-ck were up-regulated, and the expression of stromal marker Vimentin and α-SMA was decreased in the interference model group (SPAN si + COM), which was close to the negative interference control group (Figure 3).

### Using Bioinformatics Analysis to Screen the miRNA and mRNA that Could Bind to lncRNA SPANXA2-OT1

Next, we used bioinformatics analysis to predict the miRNAs that could bind to lncRNA SPANXA2-OT1. The results showed that miR-133, miR-9, miR-204, miR-214, miR-216, miR-338, miR-375, miR-214, miR-93, miR-153, miR-194, miR-196, miR-203, and miR-383 could bind to the lncRNA in the corresponding regions. Among them, miR-9, miR-203, and miR-214 were associated with the promotion of EMT, while miR-194, miR-153, miR-204, miR-338, and miR-375 were associated with its inhibition. MiR-204 and miR-194 were found to be miRNAs with high renal specificity (17). Thus, miR-204 and miR-194 were selected for further analysis.

**Figure 4:**
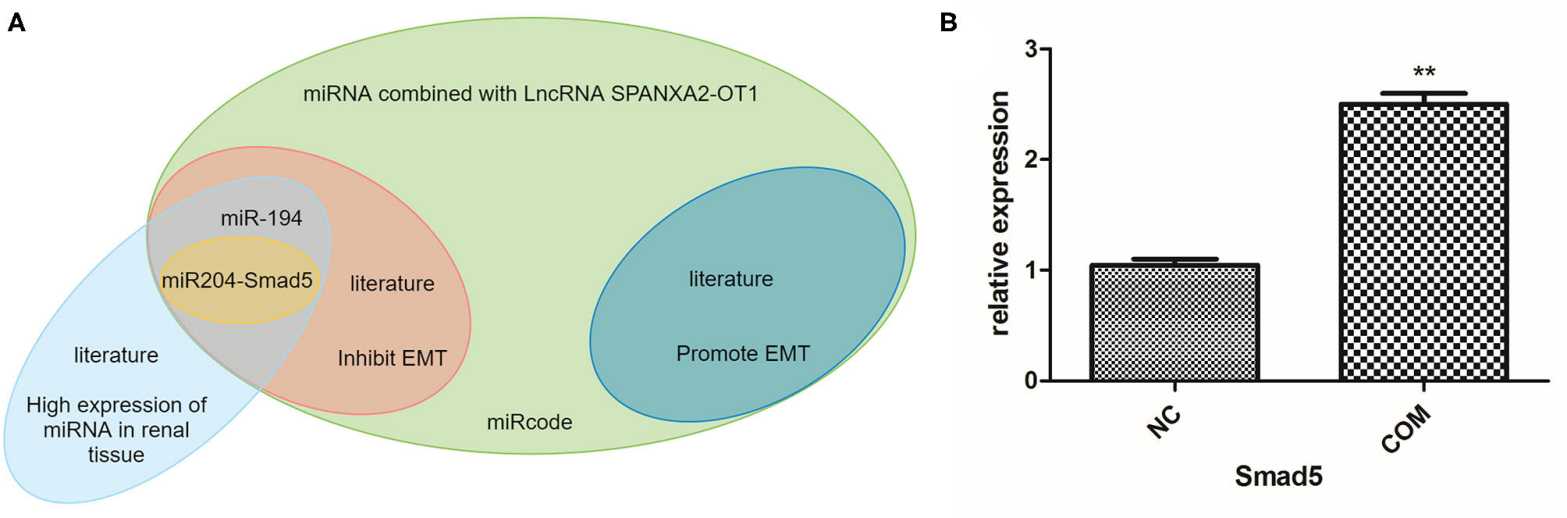
**(A)** Prediction of miRNA and mRNA that can bind to lncRNA SPANXA2-OT1. **(B)** The relative expression level of Smad5, NC, normal control group; COM, calcium oxalate monohydrate stimulated group, **p < 0.01 vs. NC group.

The most classical signaling pathway involved in renal fibrosis is TGF-/Smads signaling pathway, and almost every member of the Smad protein family has been reported to be involved in the occurrence of certain organ fibrosis (18–21). Therefore, we used bioinformatics to predict whether the target miRNA could bind to each Smad protein, finding that miR-204 could bind to Smad5 (Figure 4A). We used RT-PCR to detect the expression of Smad5 in HK-2 cells before and after COM stimulation for 48 h. The results indicated that the expression of Smad5 was significantly up-regulated after COM stimulation, as shown in Figure 4B. Therefore, we further selected miR-204 and Smad5 as the focus of our next mechanism study.

### The Role of miR-204 and Smad5 in Regulating the Occurrence of EMT

#### Using Small Interfering RNAs and miR-204 Inhibitors to Verify Whether Smad5 Was Involved in Regulating the Occurrence of EMT

**Figure 5:**
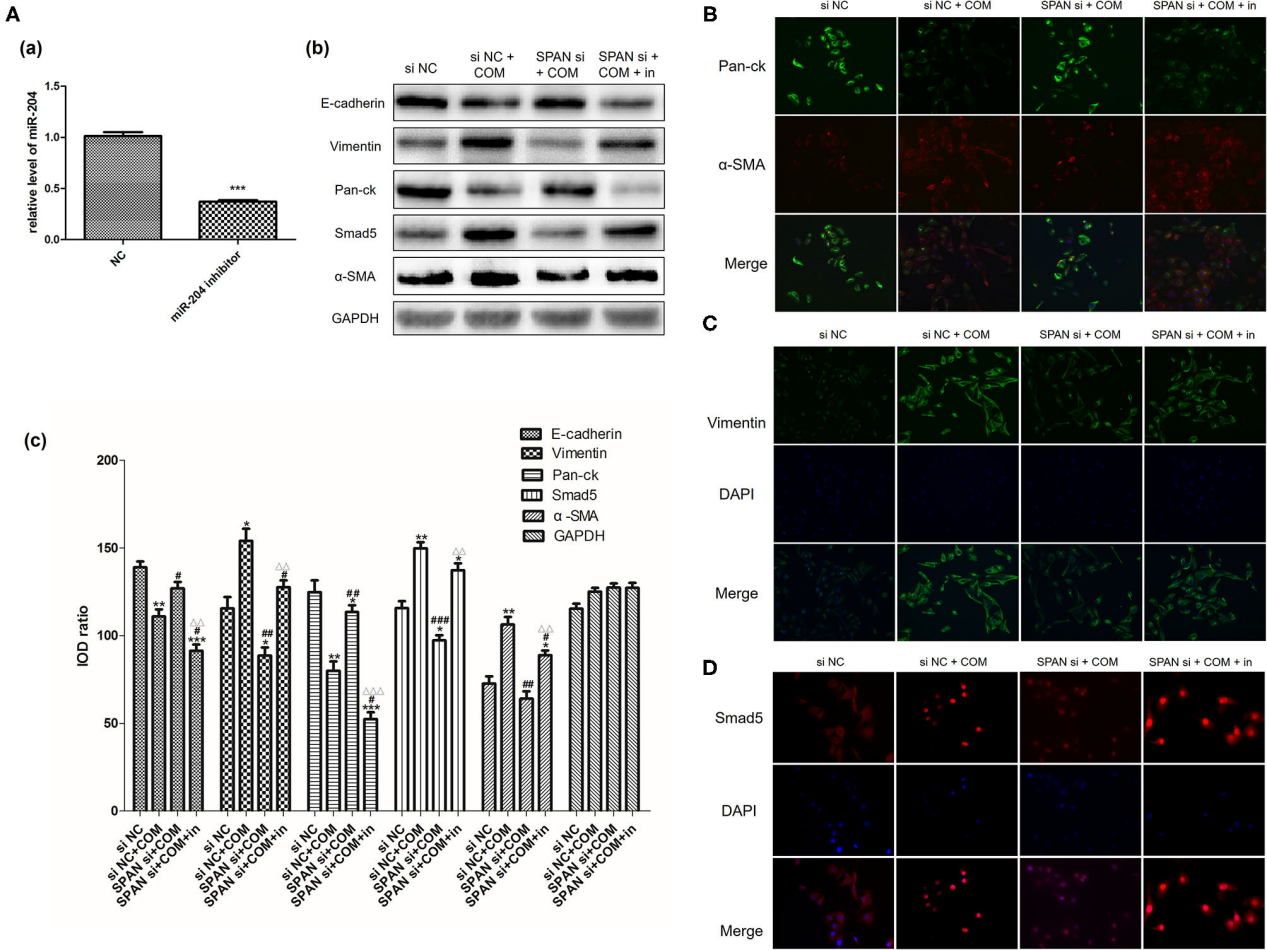
**(A)** Verification of the inhibition efficiency of miR-204 inhibitor [(a) NC, normal control group; miR-204 inhibitor, miR204 inhibition group; ***p < 0.0001 vs. NC group]; Relative expression levels of each protein in each group [(b) Western blot, (c) Western blot semi-quantitative]. siNC, negative interference control group; siNC + COM, negative interference model group; SPAN si + COM, interference model group; SPAN si + COM + in, inhibition group. *p < 0.05 vs. siNC group, **p < 0.01 vs. siNC group, ***p < 0.001 vs. siNC group, ^#^*p* < 0.05 vs. siNC + COM group, ^##^*p* < 0.01 vs. siNC + COM group, ^###^*p* < 0.001 vs. siNC + COM group, ^ΔΔ^*p* < 0.01 vs. SPAN si+COM group, ^ΔΔΔ^*p* < 0.001 vs. SPAN si+COM group. **(B)** Results of Immunofluorescence staining cells in each group (Pan-ck, green; α-SMA, red; 200x). siNC, negative interference control group; siNC + COM, negative interference model group; SPAN si + COM, interference model group; SPAN si + COM + in, inhibition model group. **(C)** Results of immunofluorescence staining cells in each group (Vimentin, green; DAPI, blue; 200x). siNC, negative interference control group; siNC + COM, negative interference model group; SPAN si + COM, interference model group; SPAN si + COM + in, inhibition group. **(D)** Results of immunofluorescence staining cells in each group (Smad5, red; DAPI, blue; 200x). siNC, negative interference control group; siNC + COM, negative interference model group; SPAN si + COM, interference model group; SPAN si + COM + in, inhibition group.

Before the experiment, we verified the inhibition efficiency of the miR-204 inhibitor by RT-PCR (Figure 5Aa). We used Western blot and immunofluorescence to detect the expression of Smad5 and the changes of EMT-related markers in each group after the addition of the miR-204 inhibitor. The results showed that the expressions of Smad5, Vimentin, and α-SMA proteins were significantly up-regulated in the negative interference model group compared with the negative interference control group, while the expressions of E-cadherin and Pan-ck were down-regulated (all *P* < 0.05). In addition, the expressions of Smad5, Vimentin, α-SMA, E-cadherin, and Pan-ck in the interference model group were close to those of the negative interference control group, while Smad5, Vimentin, α-SMA expressions of the inhibition group with miR-204 inhibitor were up-regulated and E-cadherin and Pan-ck expressions were down-regulated compared with the interference model group (all *P* < 0.05). These data suggested that the miR-204 inhibitor could alleviate the interference effect of small interfering RNA on lncRNA SPANXA2-OT1, increase the expression of Smad5, and promote the occurrence and development of EMT in renal tubular epithelial cells (Figures 5Ab–D).

#### LncRNA Overexpressed Virus and miR-204 Simulant to Verify Whether Smad5 Is Involved in Regulation of EMT

**Figure 6:**
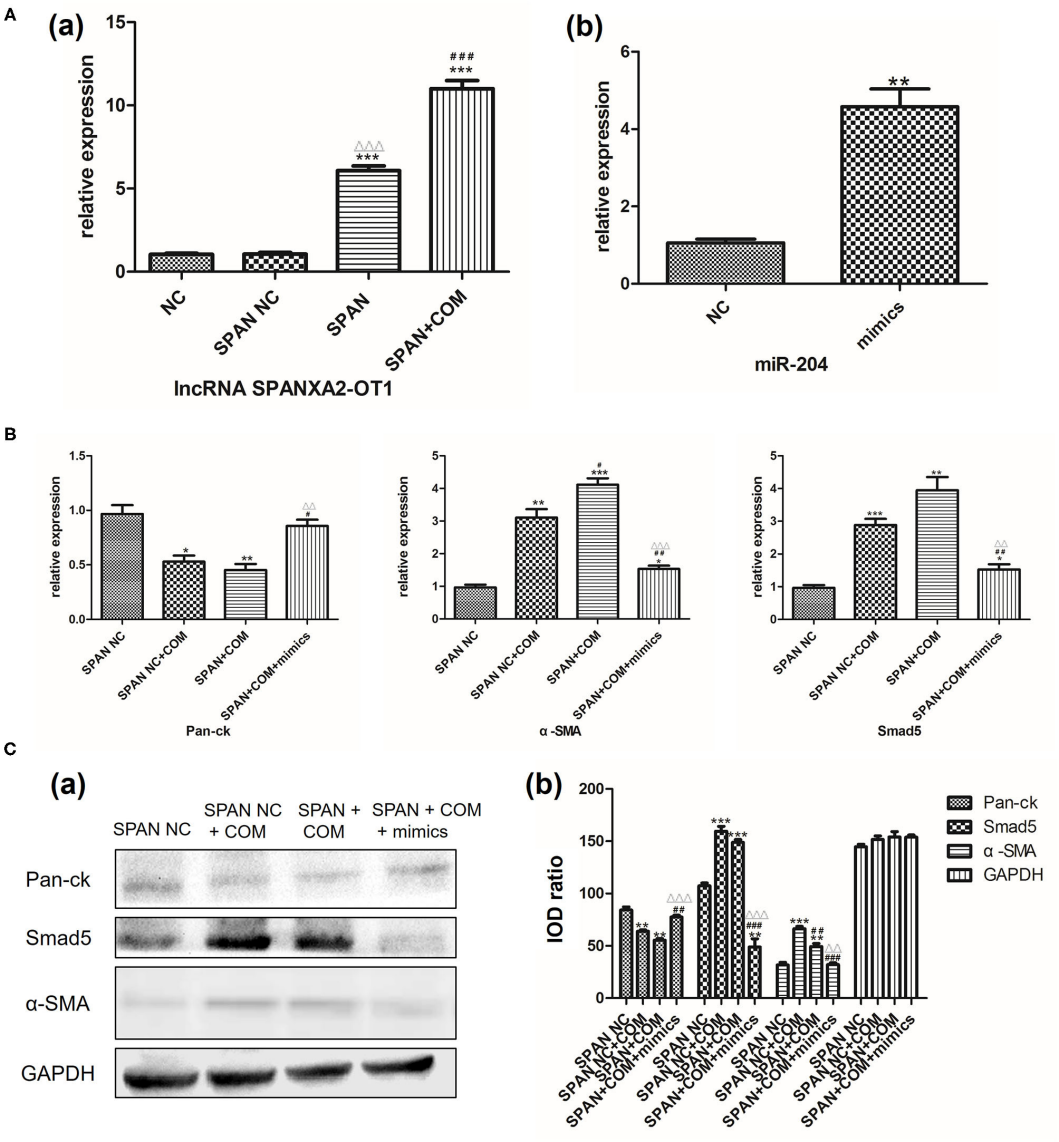
**(A)** (a) NC, normal control group; SPAN NC, null virus control group; SPAN, overexpression group; SPAN + COM, overexpression model group; ***p < 0.001 vs. NC group, ^ΔΔΔ^*p* < 0.001 vs. SPAN NC group, ^###^*p* < 0.001 vs. SPAN group. (b) NC, normal control group; mimics, miR204 simulation group; **p = 0.0017 vs. NC group. **(B)** Relative mRNA expression levels of each group. SPAN NC, null virus control group; SPAN NC + COM, null virus model group; SPAN + COM, overexpression model group; SPAN + COM + mimics, simulation group.* <0.05 vs. SPAN NC group, ** <0.01 vs. SPAN NC group, *** <0.001 vs. SPAN NC group, ^#^*p* < 0.05 vs. SPAN NC + COM group, ^##^*p* < 0.01 vs. SPAN NC + COM group, ^ΔΔ^*p* < 0.01 vs. SPAN +COM group, ^ΔΔΔ^*p* < 0.001 vs. SPAN +COM group. **(C)** Relative expression levels of each protein in each group [(a) Western blot, (b) Western blot semi-quantitative]. SPAN NC, null virus control group; SPAN NC + COM, null virus model group; SPAN + COM, overexpression model group; SPAN + COM + mimics, simulation group; **p < 0.01 vs. SPAN NC group, ***p < 0.001 vs. SPAN NC group, ^##^*p* < 0.01 vs. SPAN + COM, ^###^*p* < 0.001 vs. SPAN + COM, ^ΔΔ^*p* < 0.01 vs. SPAN + COM group, ^ΔΔΔ^*p* < 0.001 vs. SPAN +COM group.

Real-time polymerase chain reaction was used to verify the stable transfection efficiency of lncRNA SPANXA2-OT1 overexpressed viruses and the simulation efficiency of miR204 simulant. The results showed that the expression level of lncRNA SPANXA2-OT1 in SPAN group was significantly increased compared with the normal control group and the null virus control group, and the expression of miR-204 in cells after adding miR-204 mimics agent significantly increased compared with the normal control group (all *P* < 0.05), suggesting that lncRNA SPANXA2-OT1 overexpression virus stably transfected HK-2 cells successfully, and miR-204 mimics can effectively increase the expression of miR-204 in cells, as shown in Figure 6A.

Next, we used RT-PCR and Western blot to detect the expression of Smad5 and the changes of EMT-related markers in each group after the addition of miR-204 mimics. The results showed that the expression of Pan-ck was down-regulated in the null virus model group compared with the null virus control group, while the expressions of Smad5 and α-SMA were significantly up-regulated (all *P* < 0.05). However, the expression levels of Pan-ck and Smad5 have no statistical difference between the overexpressed lncRNA SPANXA2-OT1 model group and the null virus model group (all *P* > 0.05). In the simulation group, after the addition of miR-204 mimics, the expression levels of Pan-ck, α-SMA, and Smad5 were between the null virus control group and the overexpressed model group, and close to the null virus control group, suggesting that miR-204 could inhibit the expression of Smad5 and alleviate the development of EMT (Figures 6B,C).

### Double Luciferase Reporter Gene Assay

**Figure 7:**
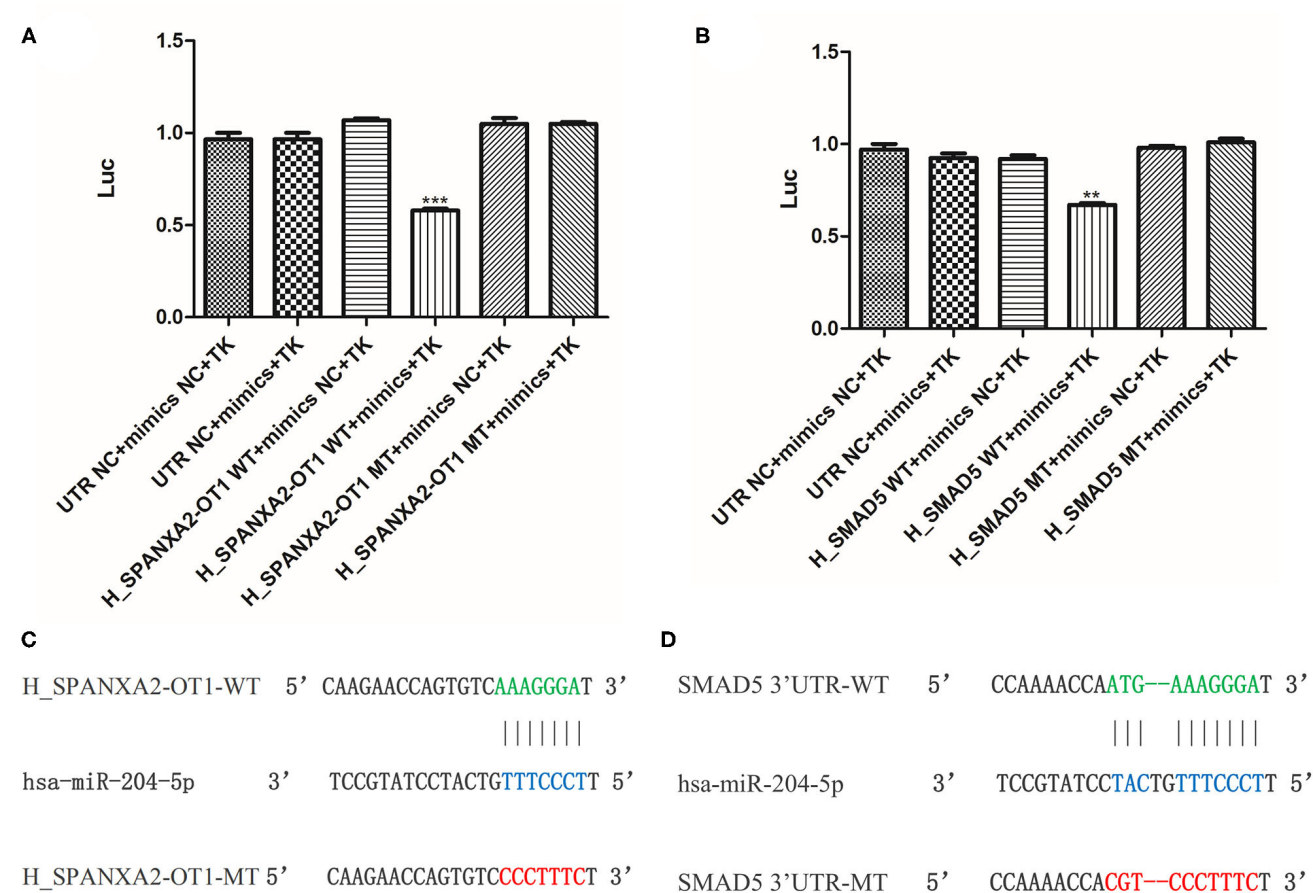
Double luciferase reporter gene assay. **(A)** Luciferase activity of 293 cells con-transfected with mimics NC, miR-204-5p, (WT) SPANXA2-OT1 and mimics NC, (WT) SPANXA2-OT1 and miR-204-5p, (MT) SPANXA2-OT1 and mimics NC, (MT) SPANXA2-OT1 and miR-204-5p for 48 h. ***p = 0.0008 vs. (WT) SPANXA2-OT1 and mimics NC. **(B)** Luciferase activity of 293 cells con-transfected with mimics NC, miR-204-5p, (WT) SMAD5 and mimics NC, (WT) SMAD5 and miR-204-5p, (MT) SMAD5 and mimics NC, (MT) SMAD5 and miR-204-5p for 48 h. **p = 0.0079 vs. (WT) SMAD5 and mimics NC. **(C)** The binding sites of the wild-type lncRNA SPANXA2-OT1 and miR-204. **(D)** The binding sites of the wild-type Smad5 and miR-204.

In order to further verify whether lncRNA SPANXA2-OT1 and Smad5 could bind to miR-204, a firefly-sea kidney dual-luciferase reporter gene assay was performed. The results showed that the transfection of miR-204-5p mimics could significantly reduce the luciferase activity of firefly carrying wild-type (WT) SPANXA2-OT1 plasmid but not the luciferase activity of firefly carrying miR-204-5p binding site mutant (MT) SPANXA2-OT1 plasmid (^***^p = 0.0008, as shown in Figure 7A). Similarly, the transfection of miR-204-5p mimics significantly reduced the luciferase activity of firemes carrying WT Smad5 plasmid but not the luciferase activity of firemes carrying miR-204-5p binding site MT Smad5 plasmid (^**^p = 0.0079, as shown in Figure 7B). The binding sites of the WT lncRNA SPANXA2-OT1 and miR-204 are shown in Figure 7C, and the binding sites of the WT Smad5 and miR-204 are shown in Figure 7D.

### Cell Proliferation and Apoptosis Experiments

**Figure 8:**
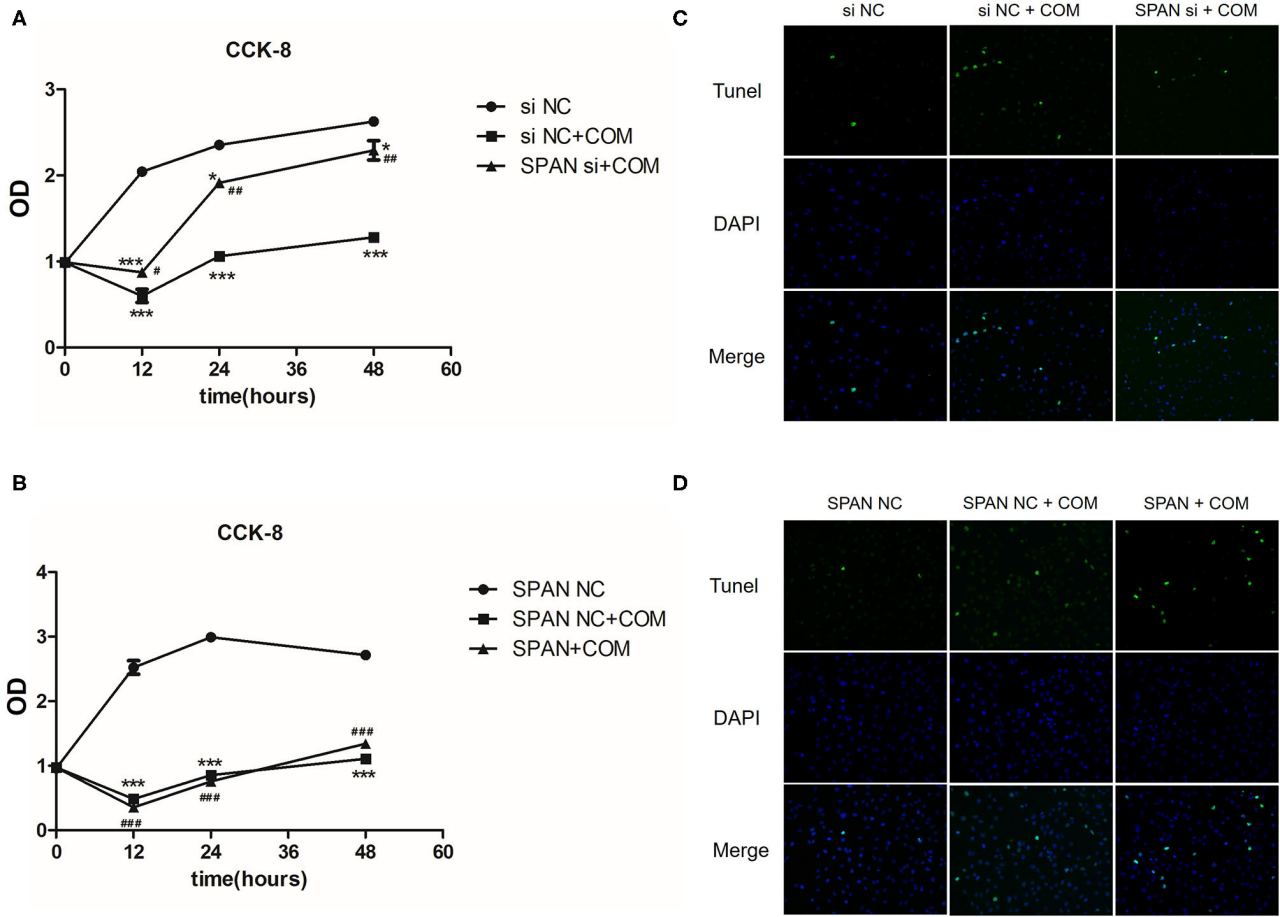
**(A)** siNC, negative interference control group; siNC + COM, negative interference model group; SPAN si + COM, interference model group; *p < 0.05 vs. siNC group, ***p < 0.0001 vs. siNC group, ^#^*p* < 0.05 vs. siNC + COM group, ^##^*p* < 0.01 vs. siNC + COM group. **(B)** SPAN NC, null virus control group; SPAN NC + COM, null virus model group; SPAN + COM, overexpression model group; ***p < 0.0001 vs. SPAN NC group, ^###^*p* < 0.0001 vs. SPAN NC group. **(C)** TUNEL test results of each group (Tunel, green; DAPI, blue; 200 x). siNC, negative interference control group; siNC + COM, negative interference model group; SPAN si + COM, interference model group. **(D)** TUNEL detection results of cells in each group (Tunel, green; DAPI, blue; 200 x). SPAN NC, null virus control group; SPAN NC + COM, null virus model group; SPAN + COM, overexpression model group.

In order to further explore whether lncRNA SPANXA2-OT1 could affect the occurrence of EMT by regulating the proliferation and apoptosis of HK-2 cells, we further improved the cell proliferation assay of CCK-8 (Figures 8A,B) and detected the apoptosis of cells in each group by TUNEL (Figures 8C,D).

#### CCK-8 Cell Proliferation Assay

The cell proliferation of the negative interference model group stimulated by COM significantly decreased compared with the negative interference control group, while the cell proliferation of the interference model group was significantly more active than that of the negative interference model group (*P* < 0.05) (Figure 8A). In addition, the cell proliferation ability of the lncRNA SPANXA2-OT1 overexpression group was significantly lower than that of the null virus control group (*P* < 0.05) (Figure 8B). These results suggested that lncRNA SPANXA2-OT1 could inhibit cell proliferation.

#### TUNEL Apoptosis Assay

The degree of apoptosis in the negative interference model group after calcium oxalate stimulation was higher than that in the negative interference control group, while the degree of apoptosis in the interference group decreased compared with the negative interference model group and was close to that in the negative interference control group (Figure 8C). In the overexpression model group, the degree of apoptosis was increased compared with the null virus control group and the null virus model control group (Figure 8D). These data suggested that lncRNA SPANXA2-OT1 could promote cell apoptosis.

## Discussion

Over recent years, obstructive nephropathy caused by urinary calculi has become one of the common factors of chronic kidney disease. Studies have found that the adhesion of urine crystals to damaged renal tubular epithelial cells is an important link in the formation of renal stones (22). In our previous study, we discovered that EMT occurred in renal tubular epithelial cells during crystalline nephropathy in the early stage of stone formation, which initiated the process of renal fibrosis (3). Progression of renal fibrosis to end-stage renal failure is a multifactorial process. Although the early occurrence of EMT in renal tubular epithelial cells has a limited impact on the final renal fibrosis (23), the damage may be irreversible if the stimulating factors are not eliminated or proper intervention measures are not taken.

In this study, we examined the mechanism of EMT during crystallinity nephropathy. We previously discovered that COM could stimulate the occurrence of EMT in HK-2 cells (4). Therefore, this model can be suitable for investigating EMT occurrence in renal tubular epithelial cells during crystallinity nephropathy. In this study, we used the same approach to further explore differentially expressed lncRNAs after COM stimulation. According to the sources of the differentially up-regulated lncRNAs (Refseq, UCSC known genes, Gencode) and fold change ≥2, eight up-regulated lncRNAs were screened. We further found that lncRNA SPANXA2-OT1 was different in HK-2 cells before and after COM stimulation for 48 h. Next, we examined whether this lncRNA was related to the occurrence and development of EMT in renal tubule epithelial cells. In order to verify the function of the screened lncRNA SPANXA2-OT1, we designed several *in vitro* experiments.

A search of the Gene Database for relevant information on lncRNA SPANXA2-OT1 showed that the lncRNA was located on the X chromosome. Due to the special genetic way that determines the X chromosome genes for certain pathological physiology, the regulation function was passed on to the next generation in a particular manner that is influenced by gender, which may explain the different incidence of kidney stones in male and female patients (24, 25).

Next, we used bioinformatics technology to predict the miRNA that could be adsorbed by this lncRNA. Sun et al. previously reported that miR-192, miR-194, miR-204, miR-215, and miR-216 in renal tissues are highly specific miRNAs (17). We further selected miR-204 and miR-194 as targets. Previous studies showed that miR-204 and miR-194 are involved in the occurrence and development of tumor-related EMT. As one of the microRNAs specifically highly expressed in the kidney, miR-204 has various biological functions. Studies have reported that miR-204-5p is involved in the occurrence and development of fibrosis in various tissues and organs and has different roles under different physiological or pathological states (26, 27). It has also been shown that miR-204-5p can regulate epithelial-mesenchymal transformation after acute renal injury induced by ischemia-reperfusion by targeting SP1 in the renal tubular epithelial cells (28). A TGF-β/Smads signaling pathway is considered the most classical signaling pathway involved in renal fibrosis; almost all members of the Smad protein family are involved in some organ fibrosis occurrence (18–21). Therefore, in the present study, we used an online bioinformatics website to predict whether the target miRNA could bind to various Smad proteins. The results suggested that miR-204 could bind to Smad5. Previous studies have shown that Smad5 can be activated by the adsorption of miR-124-3p by HOXA11-AS and then participate in the generation of keloids (29). Some studies have also shown that it is involved in the occurrence and development of liver fibrosis (30).

To further verify whether miR-204 and Smad5 are involved in the occurrence and development of EMT in HK-2 cells stimulated by COM by lncRNA SPANXA2-OT1, we used small interfering RNAs and overexpressed viruses, as well as miR-204 inhibitors and simulators. The results showed that miR-204 inhibitor could partially eliminate the inhibitory effect on the occurrence and development of EMT caused by the down-regulation of lncRNA SPANXA2-OT1, promoting EMT, and increasing the expression of Smad5. This result was also confirmed by overexpression experiments. The reporter gene assay confirmed that both the WT lncRNA SPANXA2-OT1 and the WT Smad5 could bind to miR-204. In addition, our functional experiments suggested that lncRNA SPANXA2-OT1 also had a certain degree of influence on the proliferation and apoptosis of HK-2 cells, and that lncRNA could inhibit cell proliferation and promote cell apoptosis.

In conclusion, our study suggested that lncRNA SPANXA2-OT1 could adsorb miR-204 through sponge-like action, thereby weakening the inhibitory effect of miR-204 on Smad5 and up-regulating the expression of Smad5. This lncRNA could also affect the proliferation and apoptosis of HK-2 cells, thus promoting EMT. At the gene level, it provided a theoretical basis for clinical prevention and treatment of the renal injury caused by crystalline nephropathy, which in turn, could reduce the risk of renal failure in patients with calculi. Future experimental and clinical studies are needed to further verify the function of lncRNA SPANXA2-OT1.

## Data Availability Statement

The original contributions generated for the study are included in the article/Supplementary Material, further inquiries can be directed to the corresponding author/s.

## Ethics Statement

The authors are accountable for all aspects of the work in ensuring that questions related to the accuracy or integrity of any part of the work are appropriately investigated and resolved. The trial was conducted in accordance with the Declaration of Helsinki (as revised in 2013). The study was approved by the Ethics Committee of Changhai Hospital.

## Author Contributions

HH and JZ carried out the studies. HH drafted the manuscript. YL participated in collecting data. JD and WC performed the statistical analysis and participated in its design. ZG guided the studies. All authors read and approved the final manuscript.

## Funding

This study was supported by the National Natural Science Foundation of China (grant numbers 81600521, 81770763, and 82070692) and the Subject Climbing Project of Changhai Hospital (grant number 2019YXK043). The funders had no role in study design, data collection and analysis, decision to publish, or preparation of the manuscript.

## Conflict of Interest

The authors declare that the research was conducted in the absence of any commercial or financial relationships that could be construed as a potential conflict of interest.

## Publisher's Note

All claims expressed in this article are solely those of the authors and do not necessarily represent those of their affiliated organizations, or those of the publisher, the editors and the reviewers. Any product that may be evaluated in this article, or claim that may be made by its manufacturer, is not guaranteed or endorsed by the publisher.

## Abbreviations

lncRNAs: long non-coding RNAs
EMT: epithelial-mesenchymal transition
COM: calcium oxalate monohydrate
RIF: renal interstitial fibrosis
qRT-PCR: quantitative real-time polymerase chain reaction
SDS-PAGE: sulfate-polyacrylamide gel electrophoresis
CCK-8: cell counting kit-8
OD: optical density
WT: wild-type
MT: mutant.
