# Supplementary material for: LncRNA SPANXA2-OT1 Participates in the Occurrence and Development of EMT in Calcium Oxalate Crystal-Induced Kidney Injury by Adsorbing miR-204 and Up-Regulating Smad5
Source: Front Med (Lausanne). 2021 Sep 27;8:719980. doi: 10.3389/fmed.2021.719980 (PMC8502877; doi:10.3389/fmed.2021.719980)
Supplement: Supplementary file 1 [file Table_1.docx]

**Supplementary Table S1.** Primer sequence of related RNA

| **Gene** | **Forward (5’→3’)** | **Reverse (5’→3’)** |
| --- | --- | --- |
| SPANXA2-OT1 siRNA1 | AAGGCTAACTACCTGAATATC | GATATTCAGGTAGTTAGCCTT |
| SPANXA2-OT1 siRNA2 | AAGCTTGCATACATGGCCACT | AGTGGCCATGTATGCAAGCTT |
| SPANXA2-OT1 siRNA3 | AAGCAAGCTAGACTTTCAACT | AGTTGAAAGTCTAGCTTGCTT |
| MiR-204 | GCGGCGCAAAGAATTCTCCT | GTGCAGGGTCCGAGGT |
| U6 | CTCGCTTCGGCAGCACA | AACGCTTCACGAATTTGCGT |
| hsa-mir-204-5p inhibitor | AGGCAUAGGAUGACAAAGGGAA |  |
| Smad5 | CCAGCAGTAAAGCGATTGTTGG | GGGGTAAGCCTTTTCTGTGAG |
| hsa-miR-204-5p mimics | UUCCCUUUGUCAUCCUAUGCCU | AGGCAUAGGAUGACAAAGGGAA |
| Pan-ck | GTCCTACAGATTGACAATGC | CACGCTCTGGATCTGTGACAG |
| α-SMA | CTGTCCCTCTATGCCTCTGG | AGGGCTGTGATCTCCTTCTG |
| β-actin | TCTACAATGAGCTGCGTGTG | GGTCAGGATCTTCATGAGGT |
