# Supplementary material for: LncRNA SPANXA2-OT1 Participates in the Occurrence and Development of EMT in Calcium Oxalate Crystal-Induced Kidney Injury by Adsorbing miR-204 and Up-Regulating Smad5
Source: Front Med (Lausanne). 2021 Sep 27;8:719980. doi: 10.3389/fmed.2021.719980 (PMC8502877; doi:10.3389/fmed.2021.719980)

SiNC s iNC+CAOX SPANsi+CAOX

1. Cad
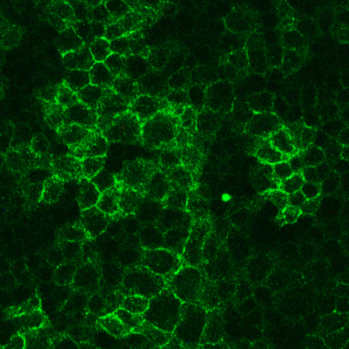

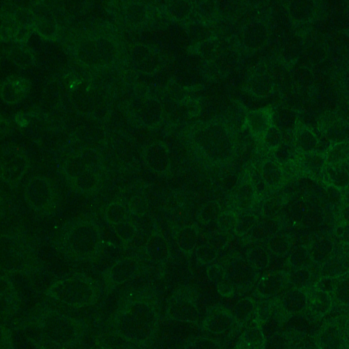

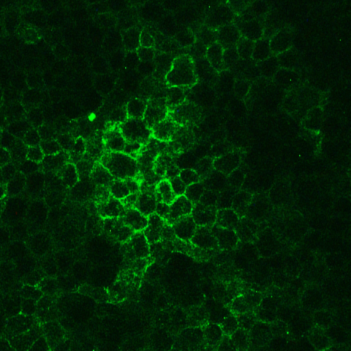


Dapi
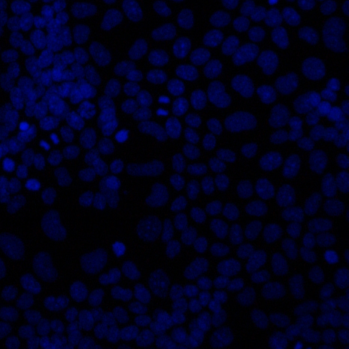

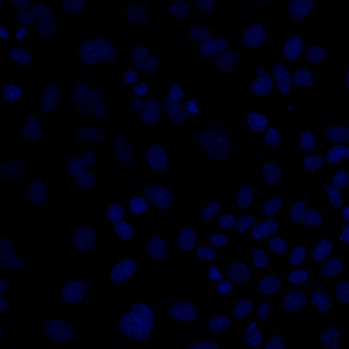

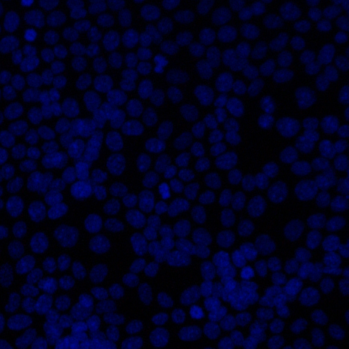


Merge
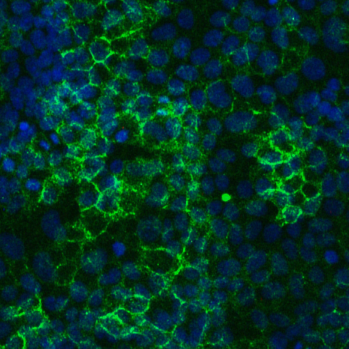

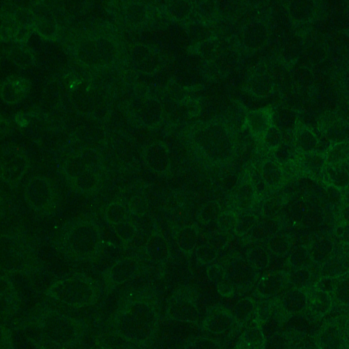

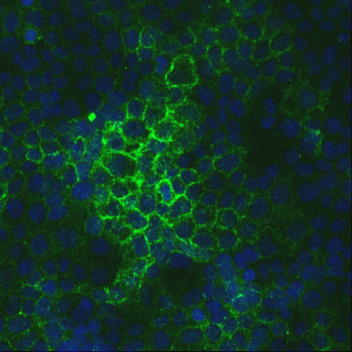


SiNC s iNC+CAOX SPANsi+CAOX

Vim
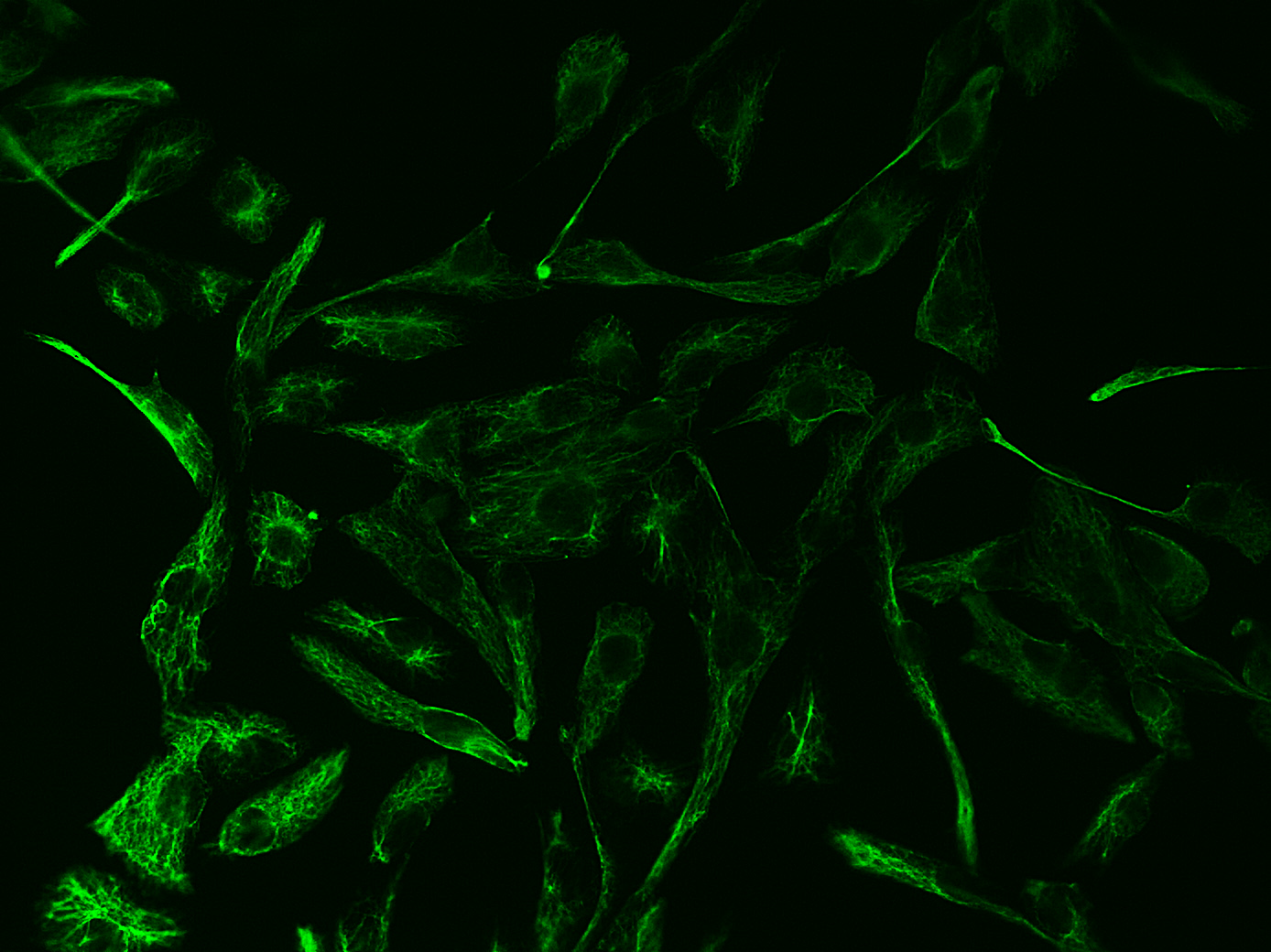

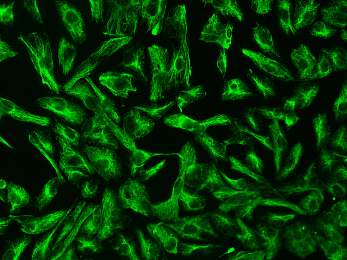

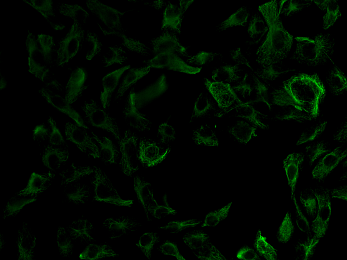


DAPI
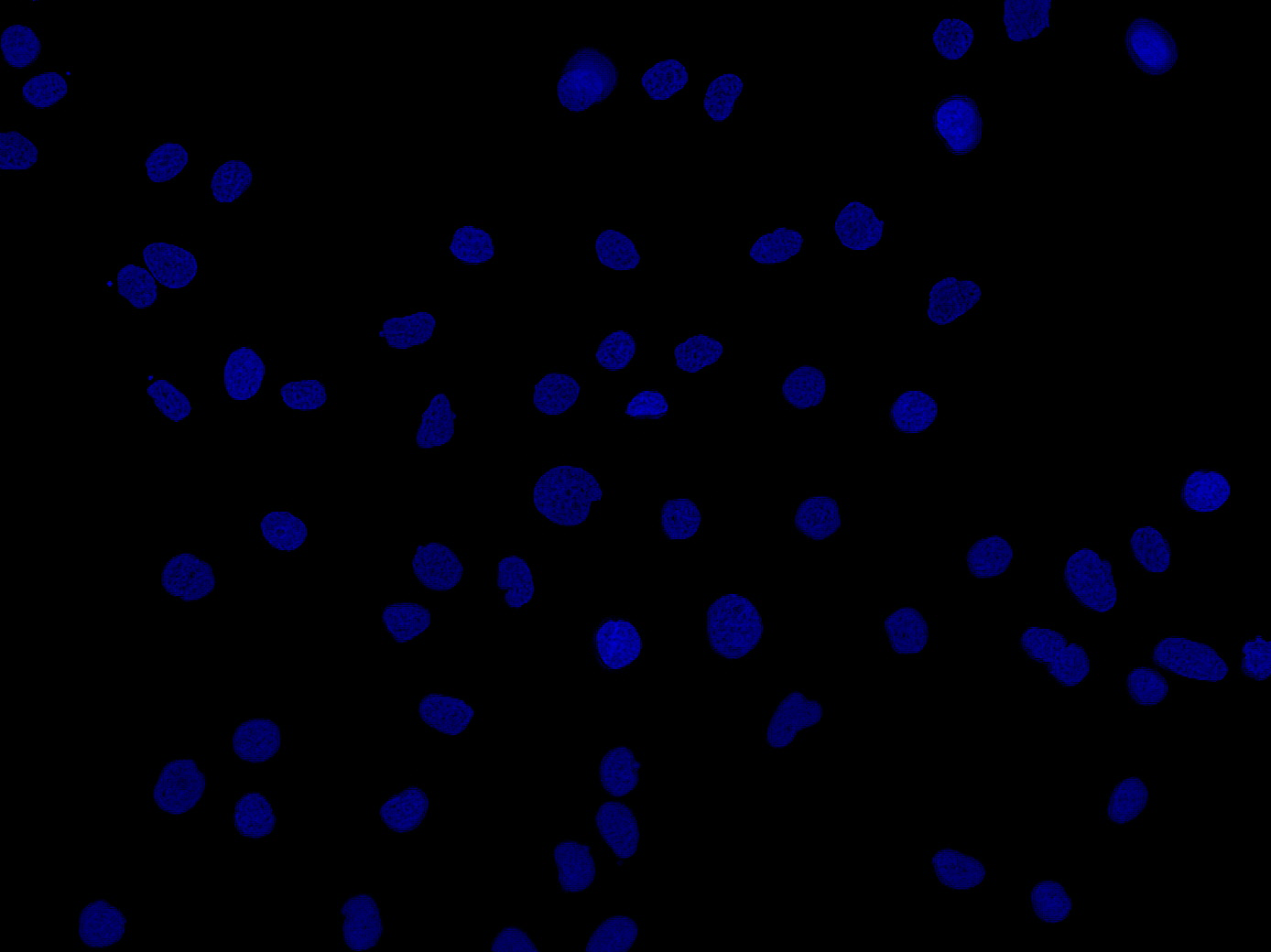

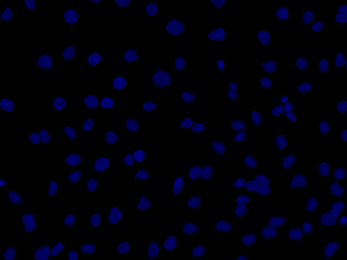

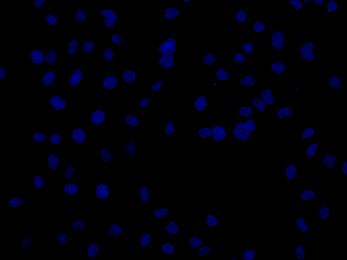


Merge
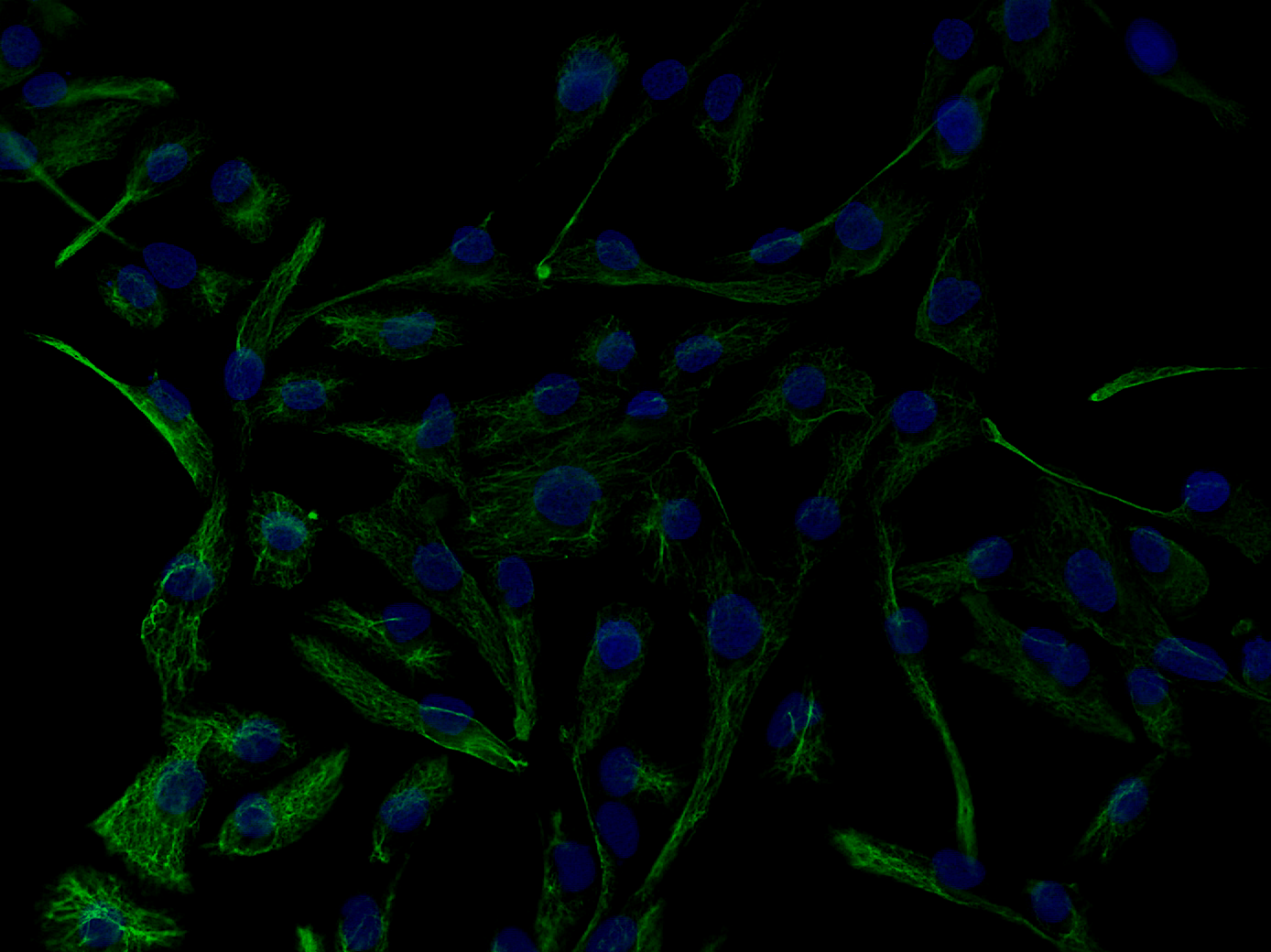

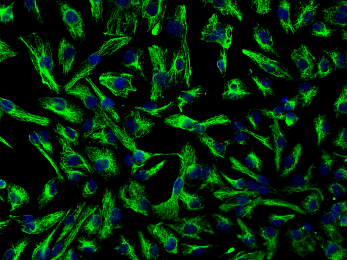

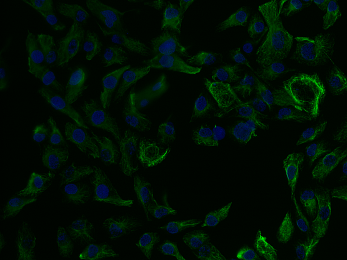


SiNC s iNC+CAOX SPANsi+CAOX

Pan-ck
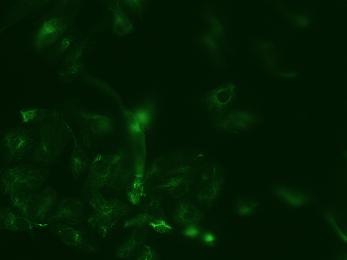

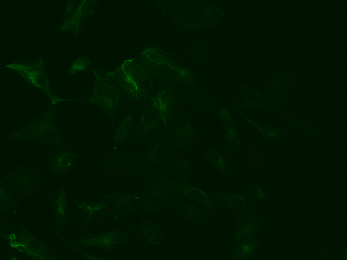

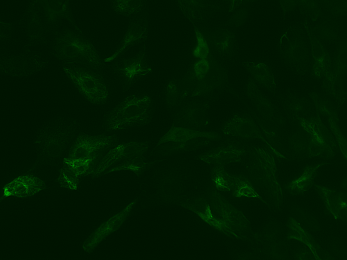


α-SMA
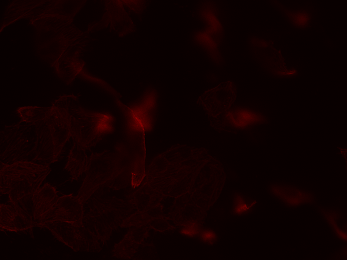

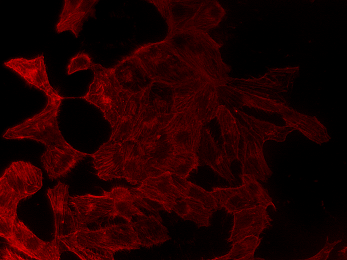

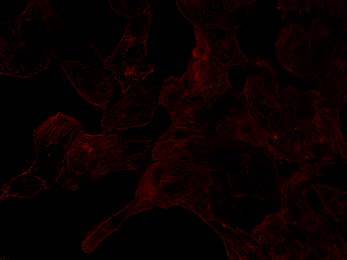


Merge
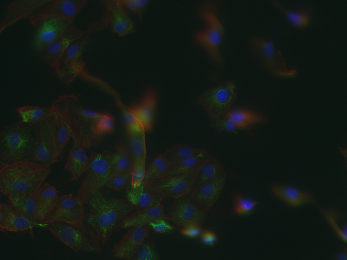

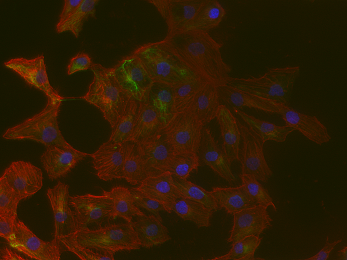

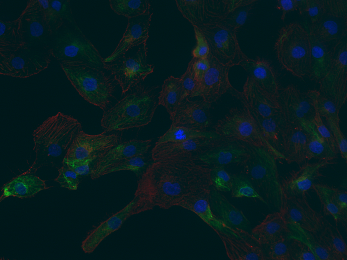

Supplement: Supplementary file 6 [file Table_6.DOCX]
