# Supplementary material for: LncRNA SPANXA2-OT1 Participates in the Occurrence and Development of EMT in Calcium Oxalate Crystal-Induced Kidney Injury by Adsorbing miR-204 and Up-Regulating Smad5
Source: Front Med (Lausanne). 2021 Sep 27;8:719980. doi: 10.3389/fmed.2021.719980 (PMC8502877; doi:10.3389/fmed.2021.719980)

siNC siNC+CAOX SPAN si+CAOX SPAN si+CAOX+in

Pan-ck
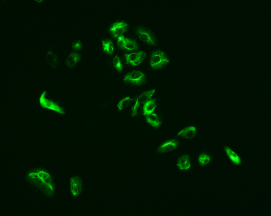

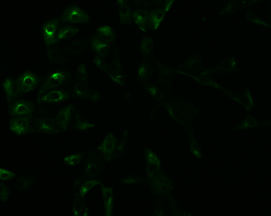

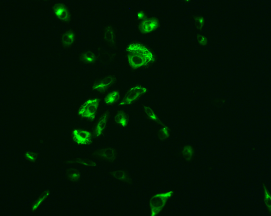

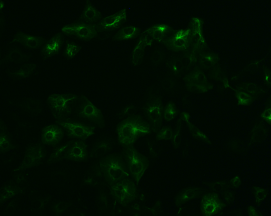


α-SMA
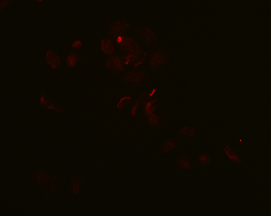

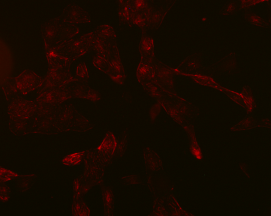

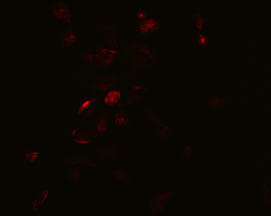

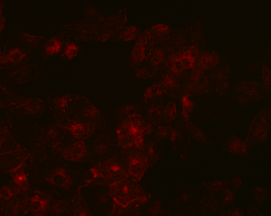


Merge
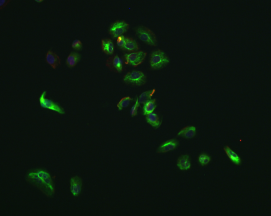

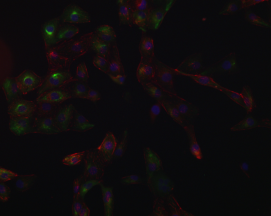

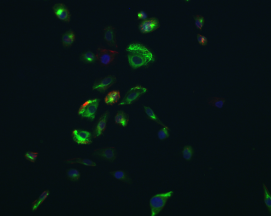

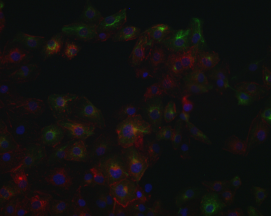


siNC siNC+CAOX SPAN si+CAOX SPAN si+CAOX+in

Vim
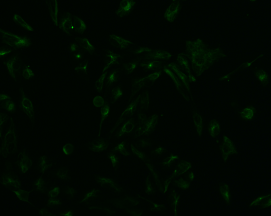

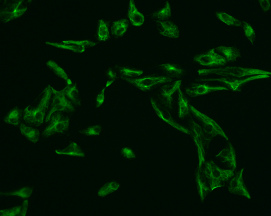

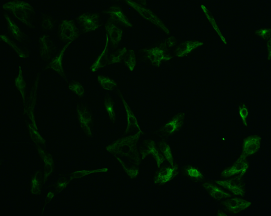

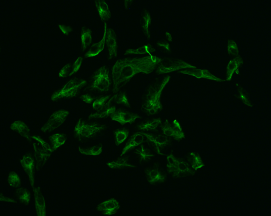


Dapi
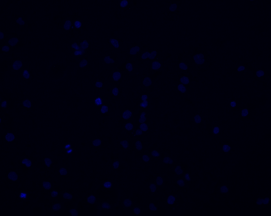

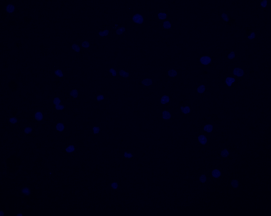

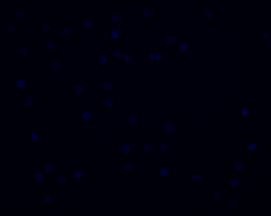

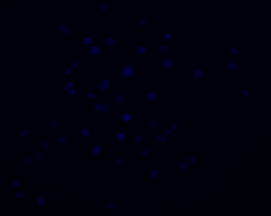


Merge
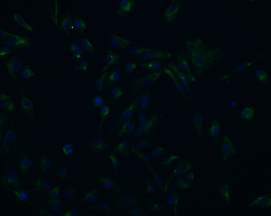

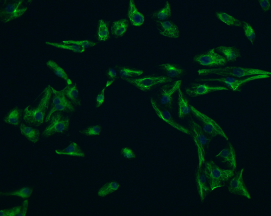

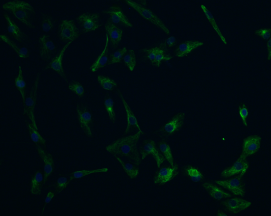

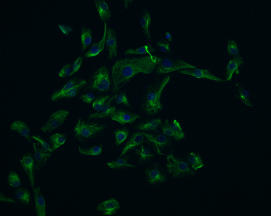


siNC siNC+CAOX SPAN si+CAOX SPAN si+CAOX+in

Smad5
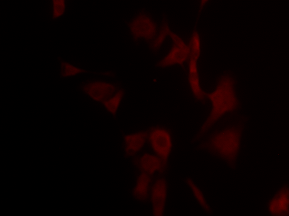

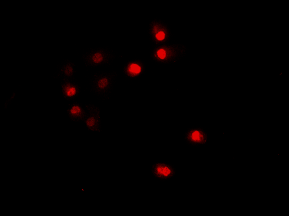

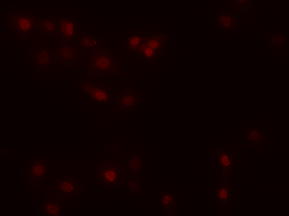

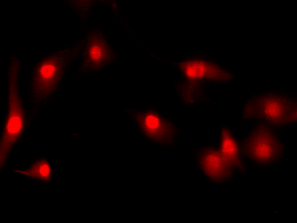


DAPI
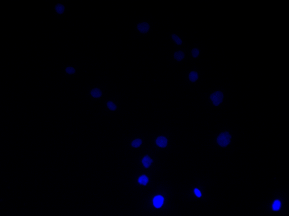

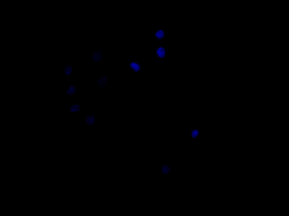

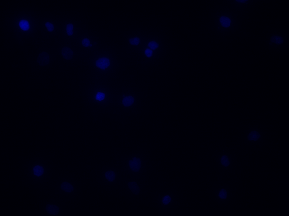

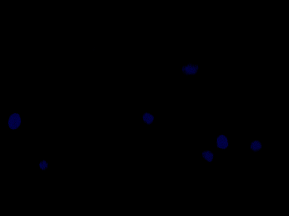


Merge
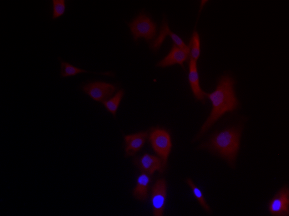

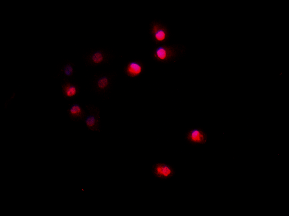

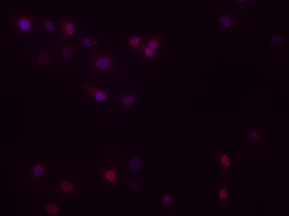

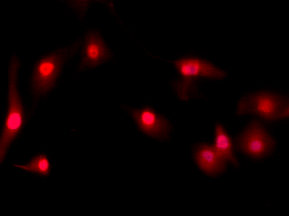

Supplement: Supplementary file 7 [file Table_7.DOCX]
