# Supplementary figures and images for: LncRNA SPANXA2-OT1 Participates in the Occurrence and Development of EMT in Calcium Oxalate Crystal-Induced Kidney Injury by Adsorbing miR-204 and Up-Regulating Smad5
Source: Front Med (Lausanne). 2021 Sep 27;8:719980. doi: 10.3389/fmed.2021.719980 (PMC8502877; doi:10.3389/fmed.2021.719980)

siNC siNC+CAOX SPAN si+CAOX

TUNEL
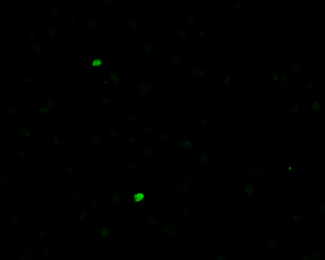

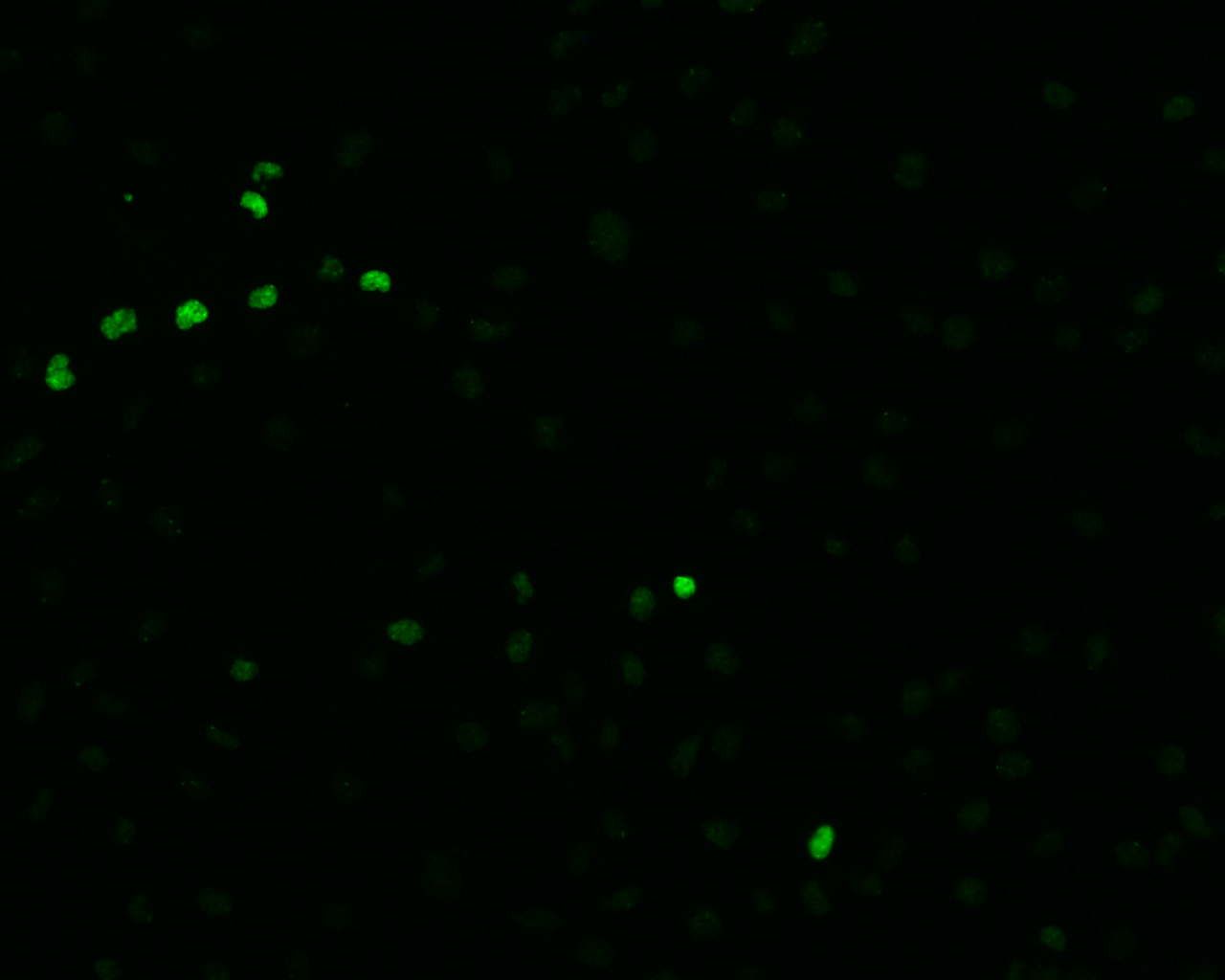

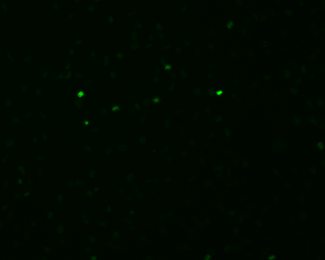


DAPI
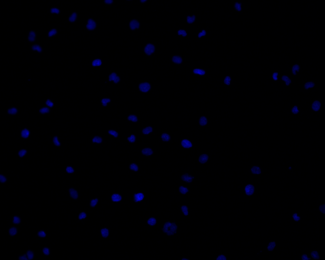

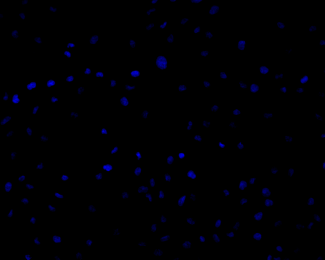

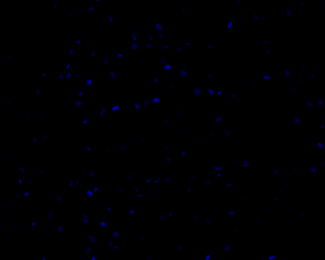


Merge
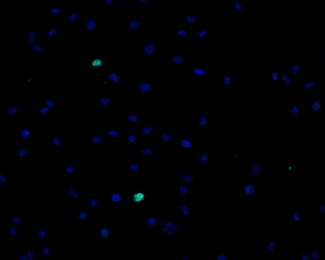

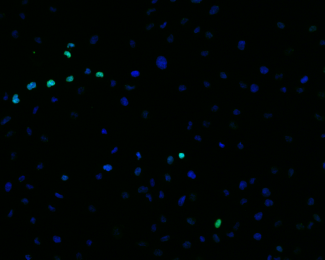

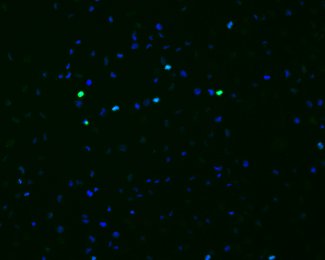


SPAN NC SPAN NC+CAOX SPAN+CAOX

TUNEL
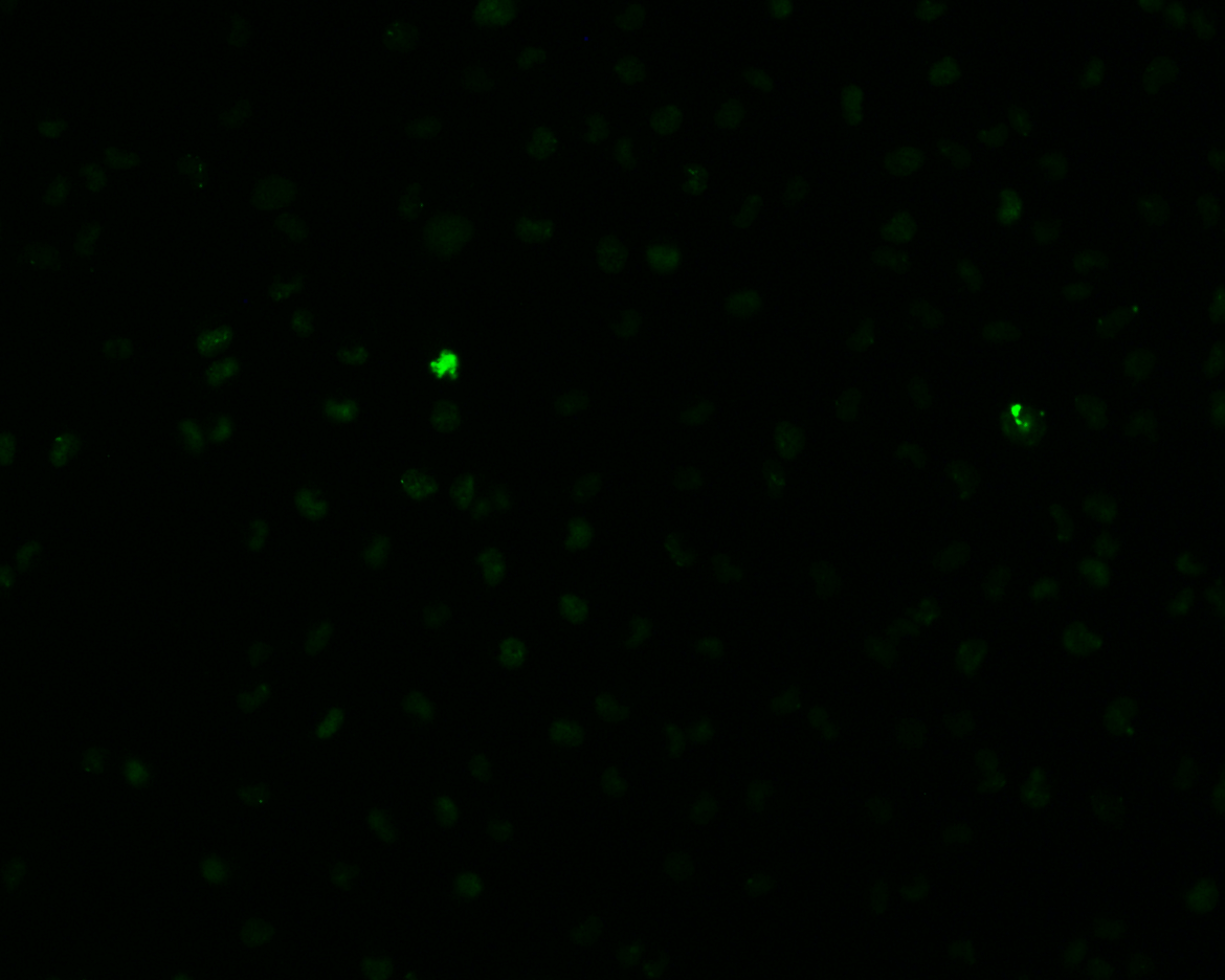

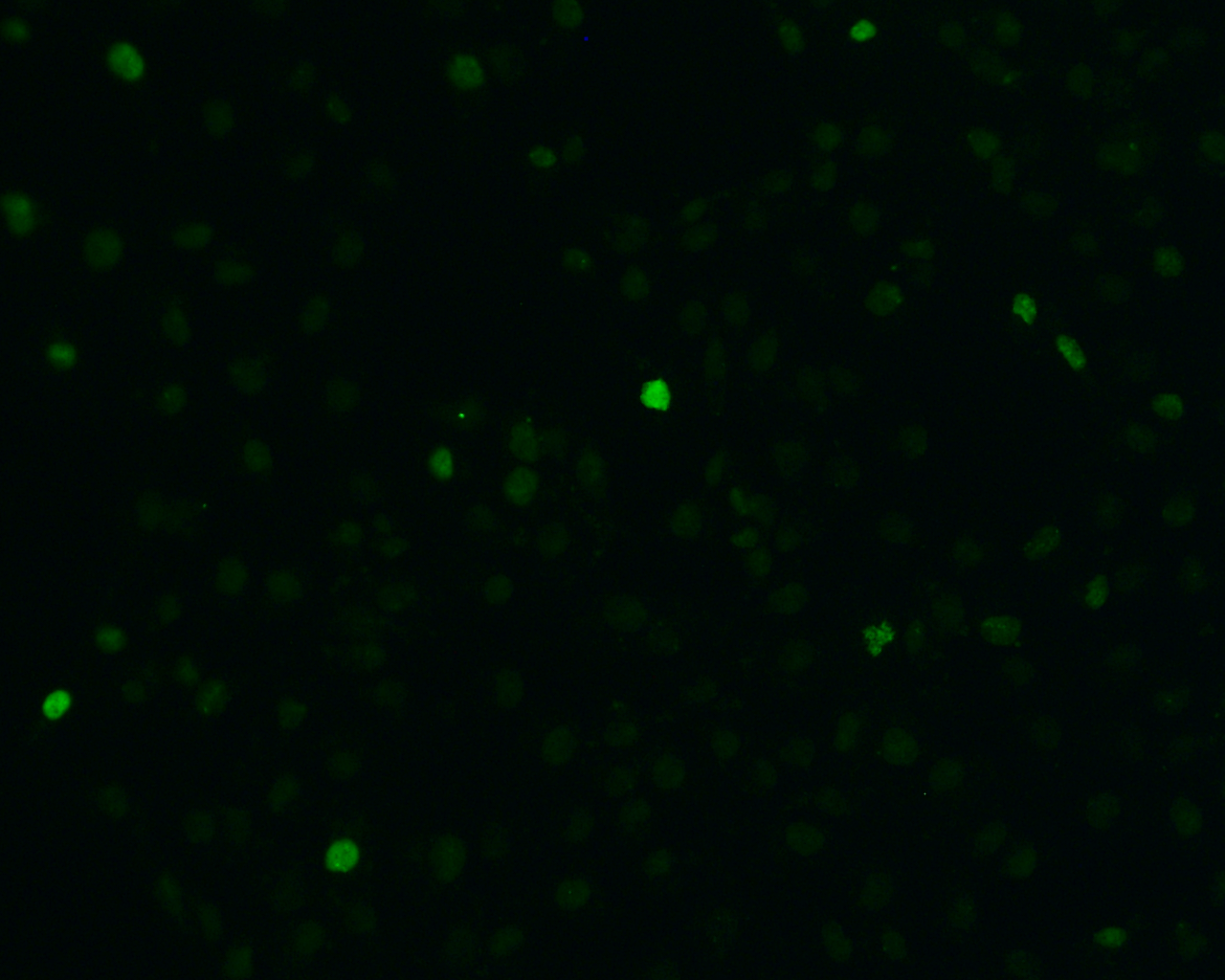

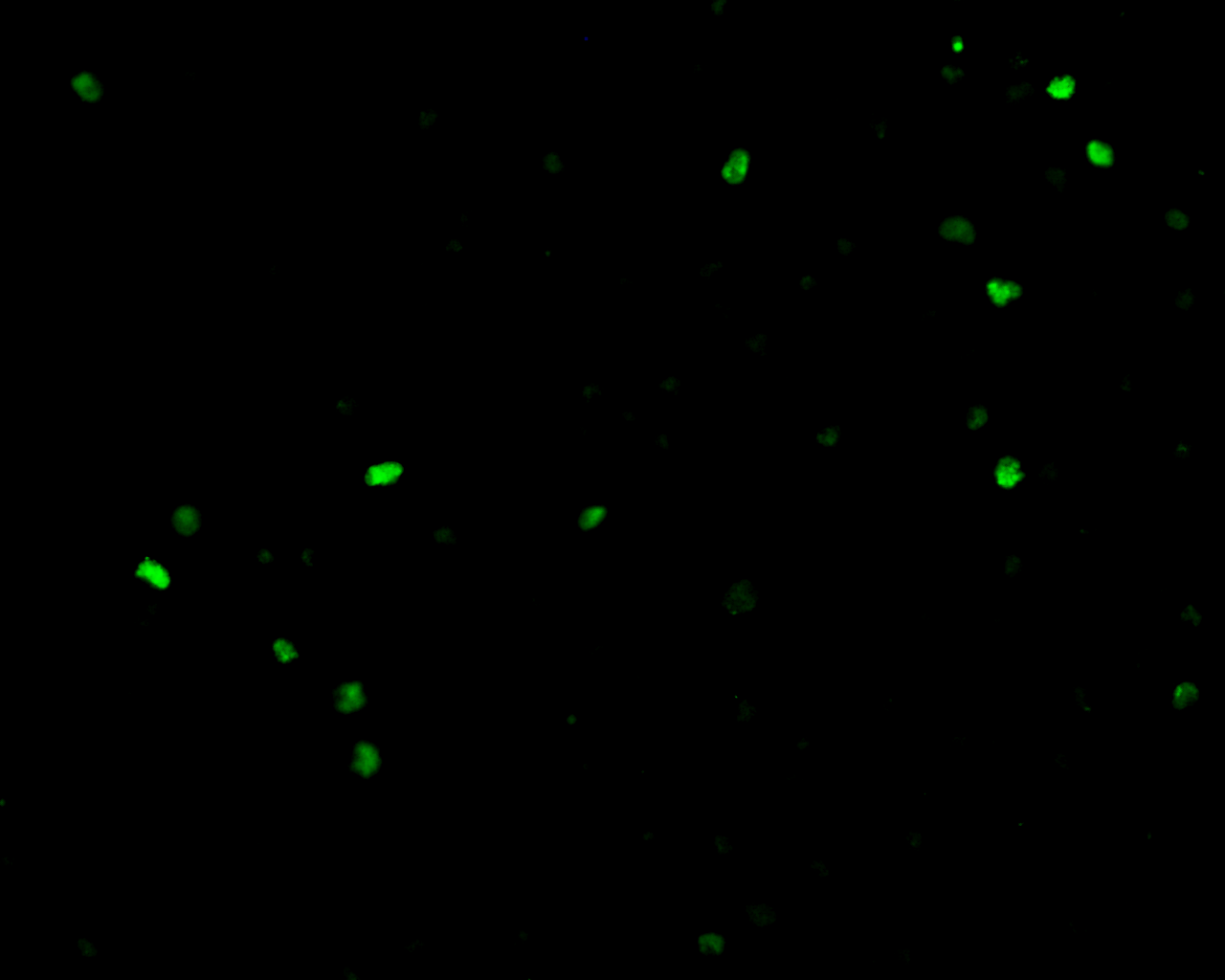


DAPI
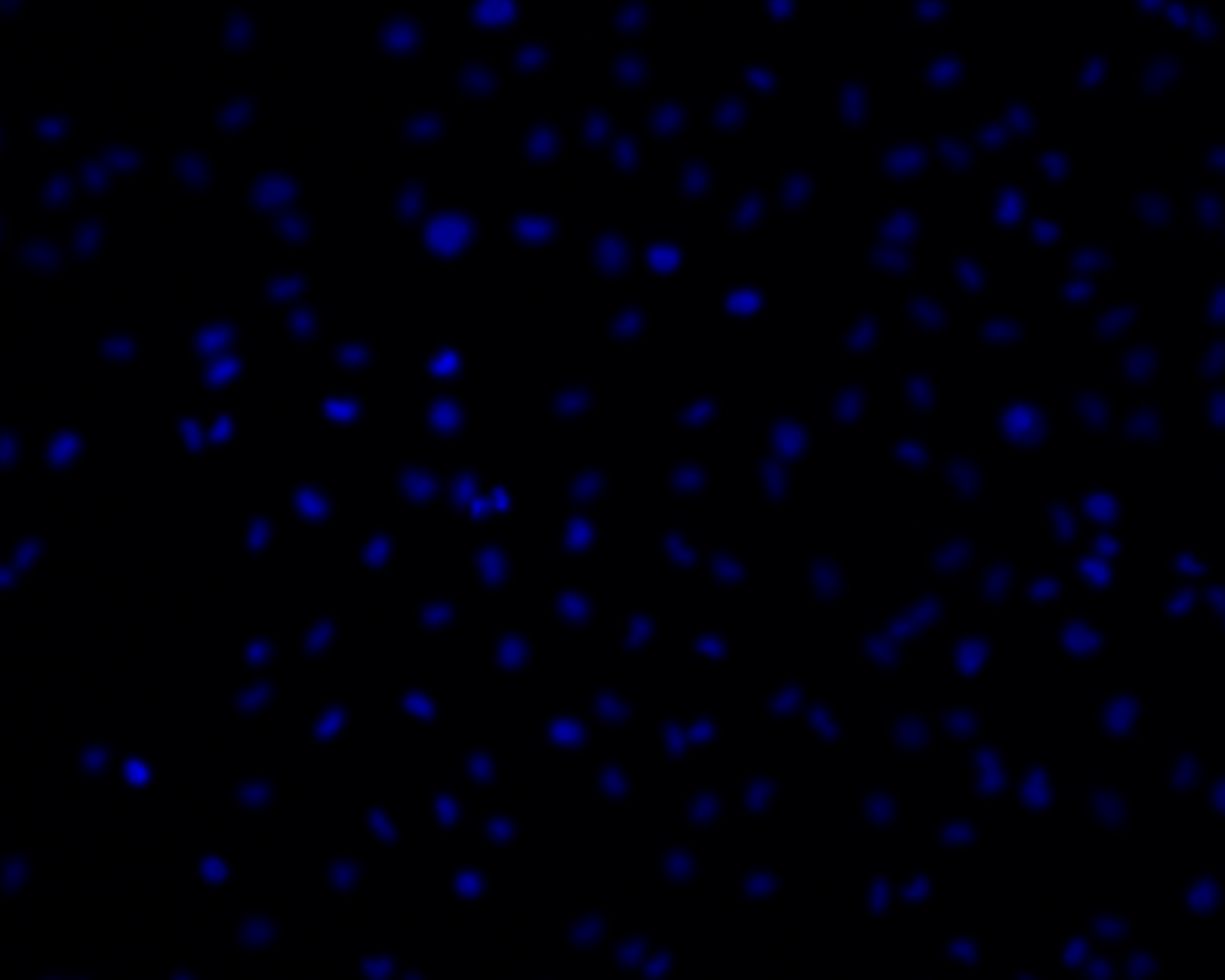

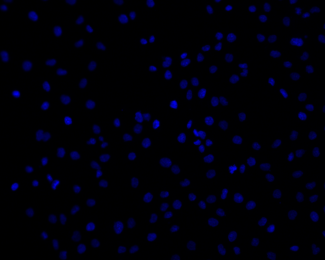

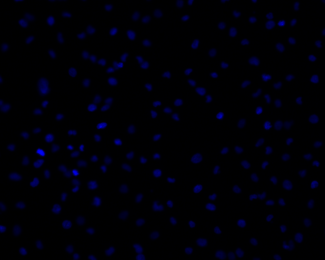


Merge
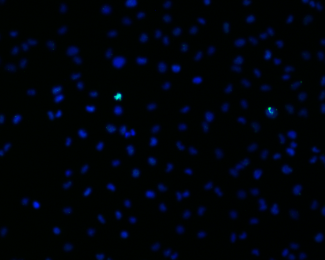

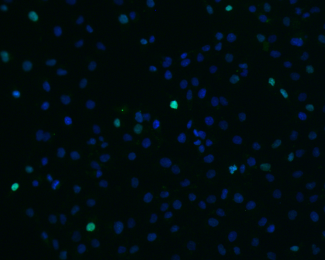

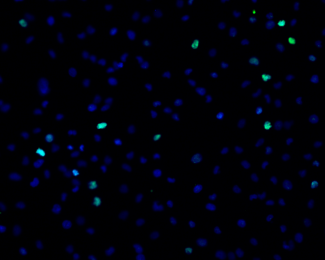

Supplement: Supplementary file 8 [file Table_8.DOCX]

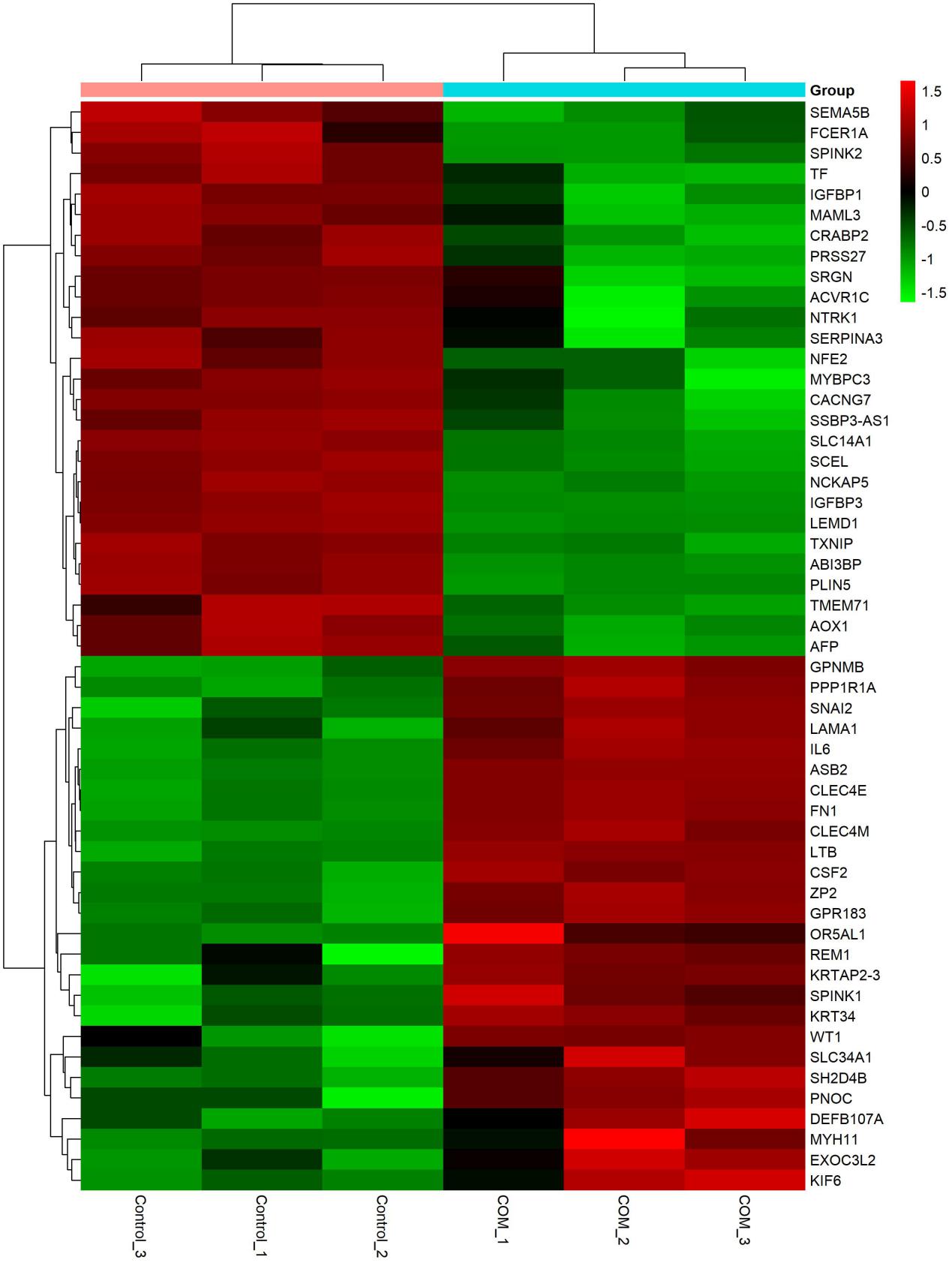

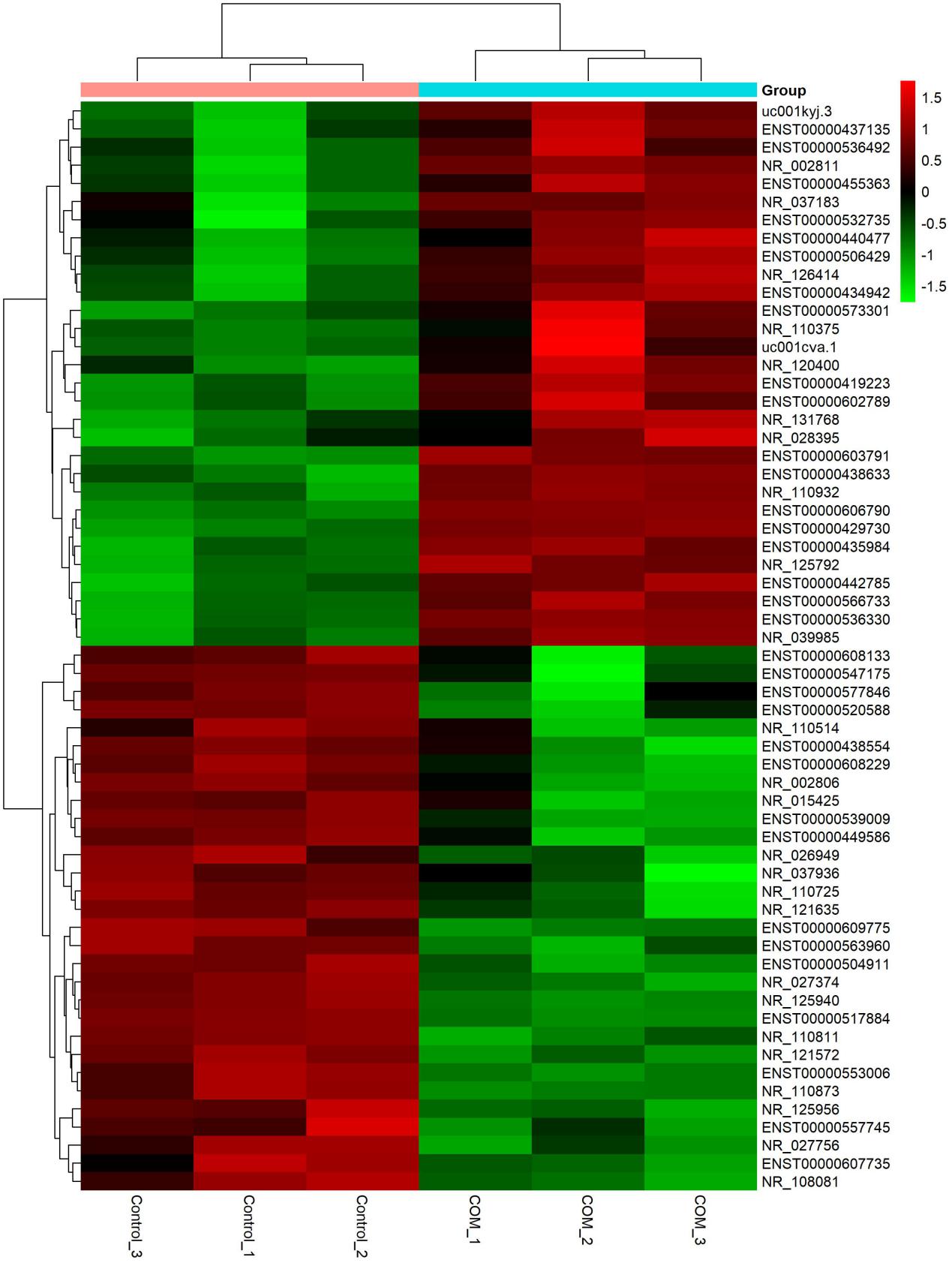

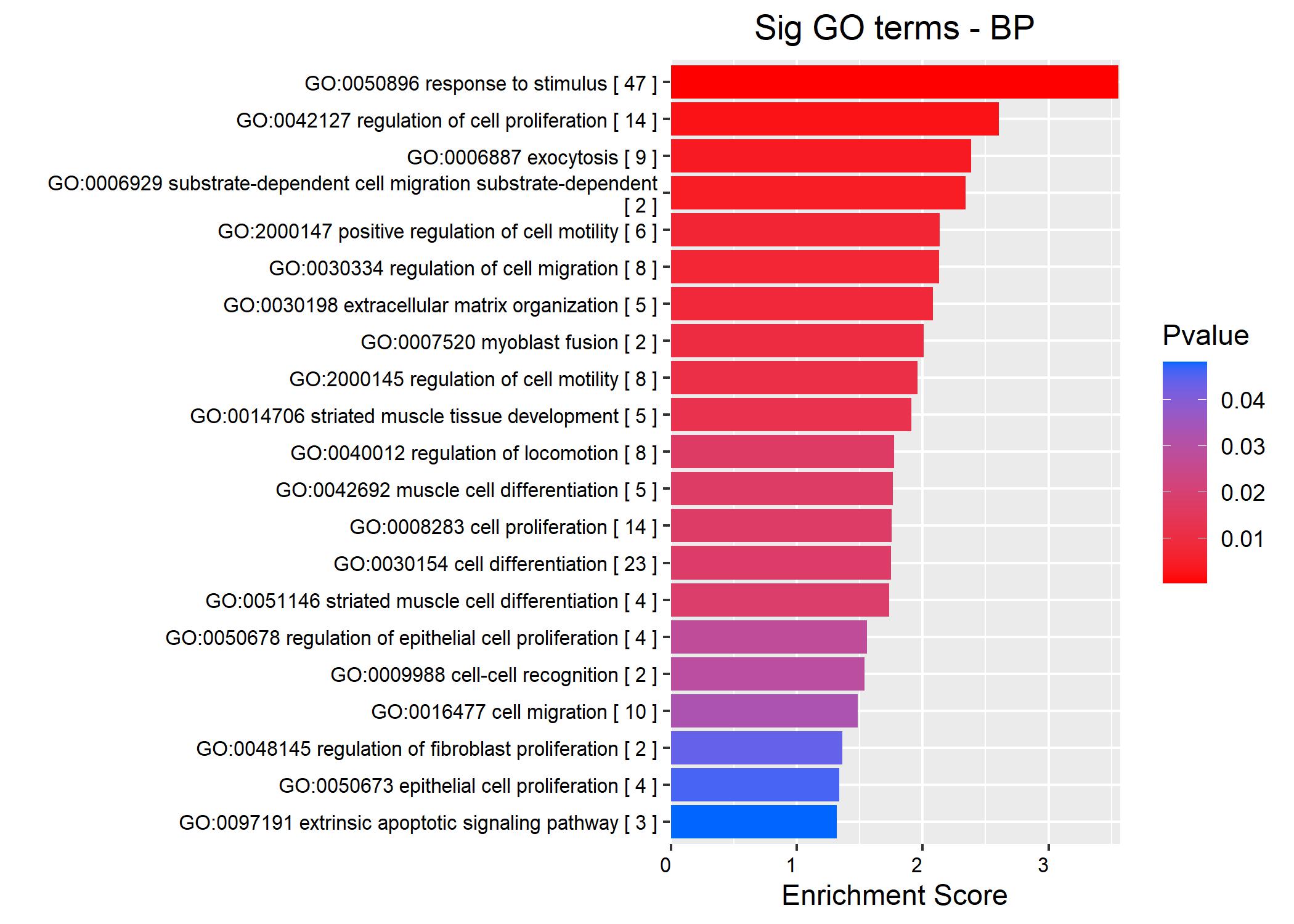


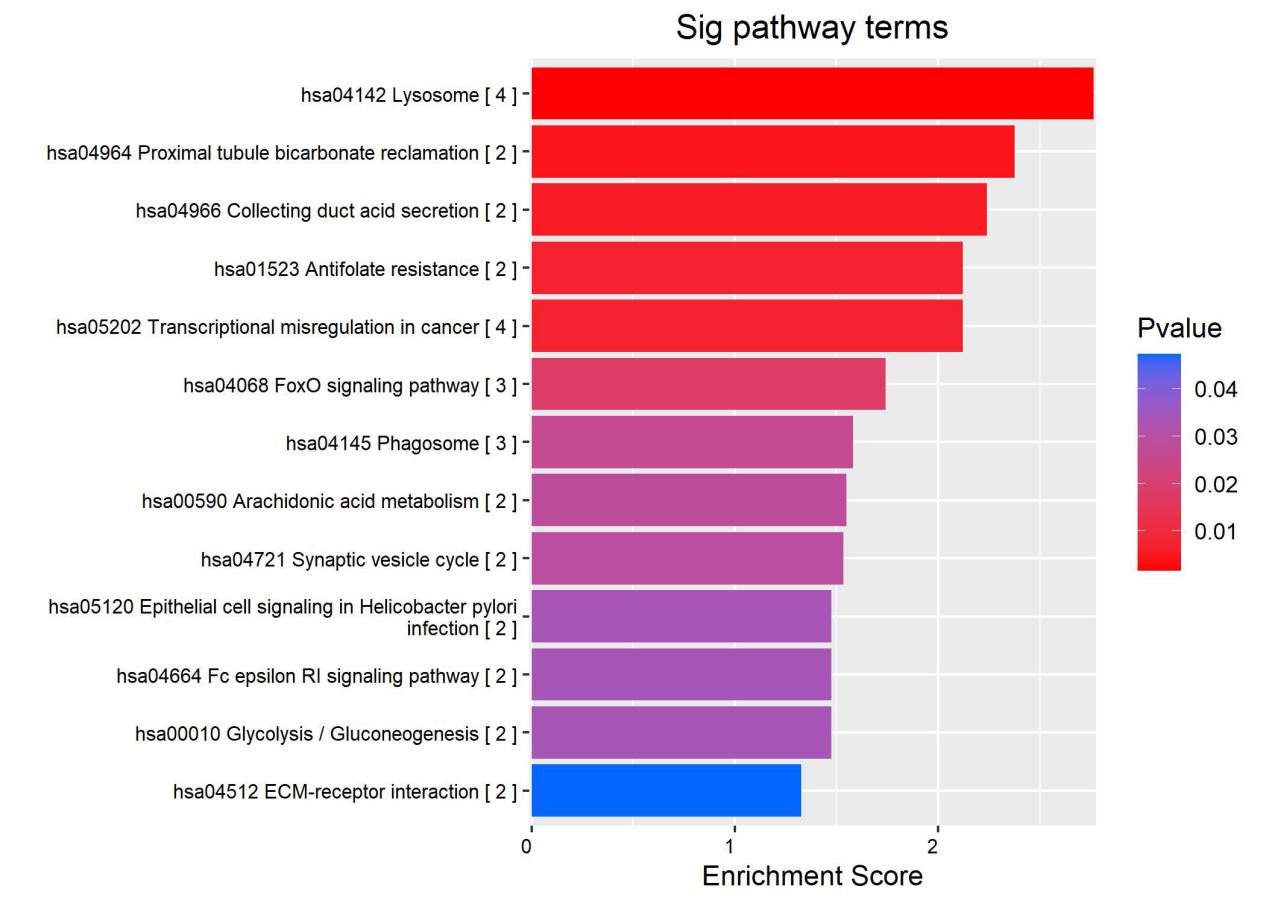

Supplement: Supplementary file 9 [file Table_9.DOCX]

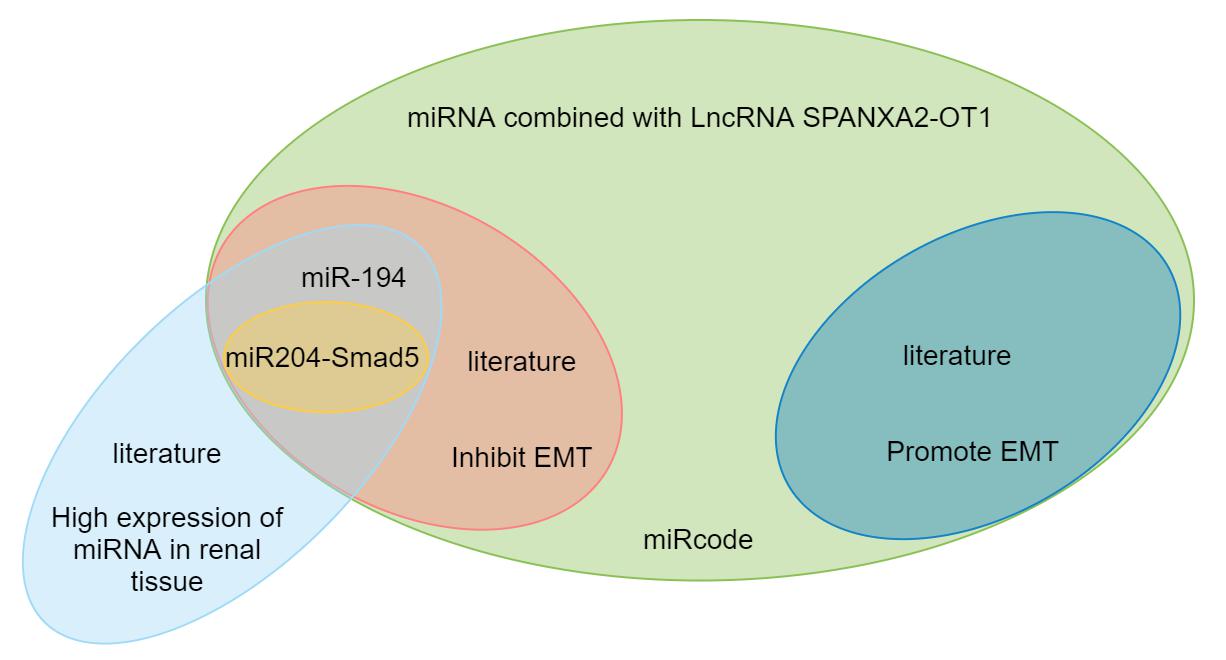


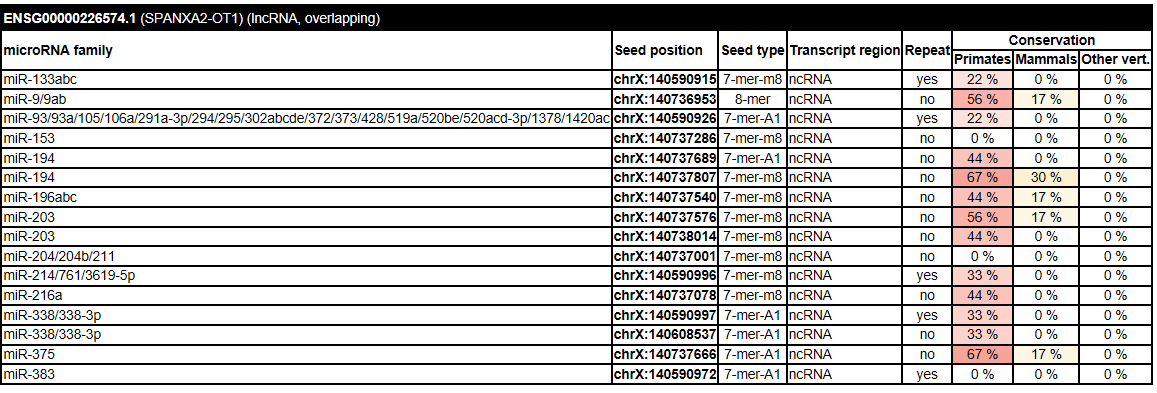


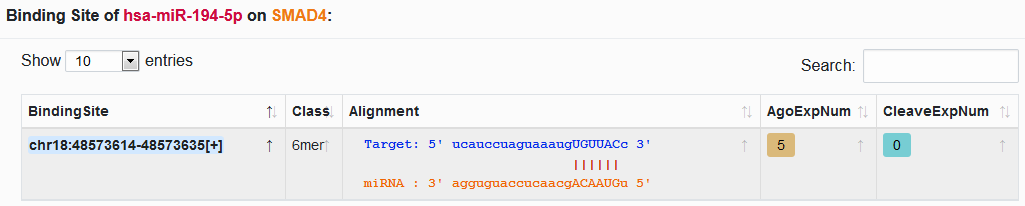


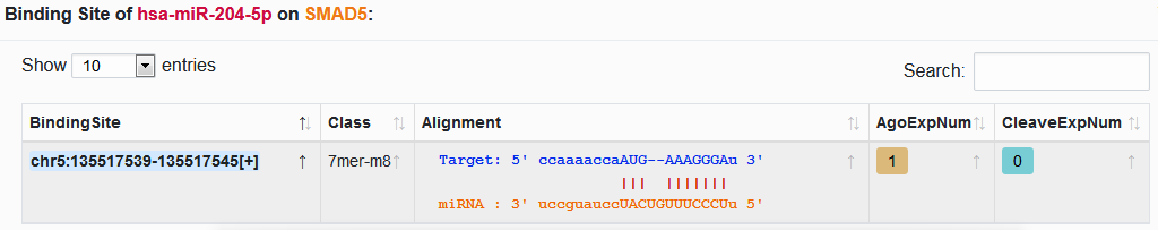

Supplement: Supplementary file 10 [file Table_10.DOCX]

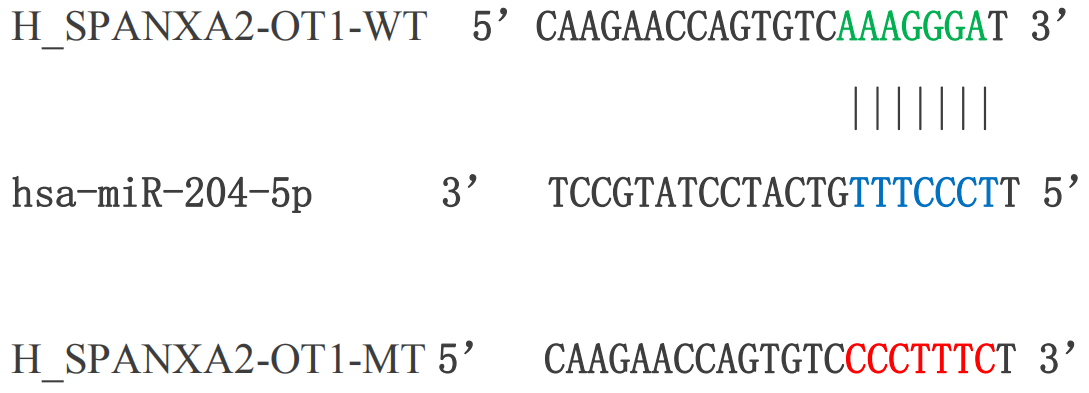


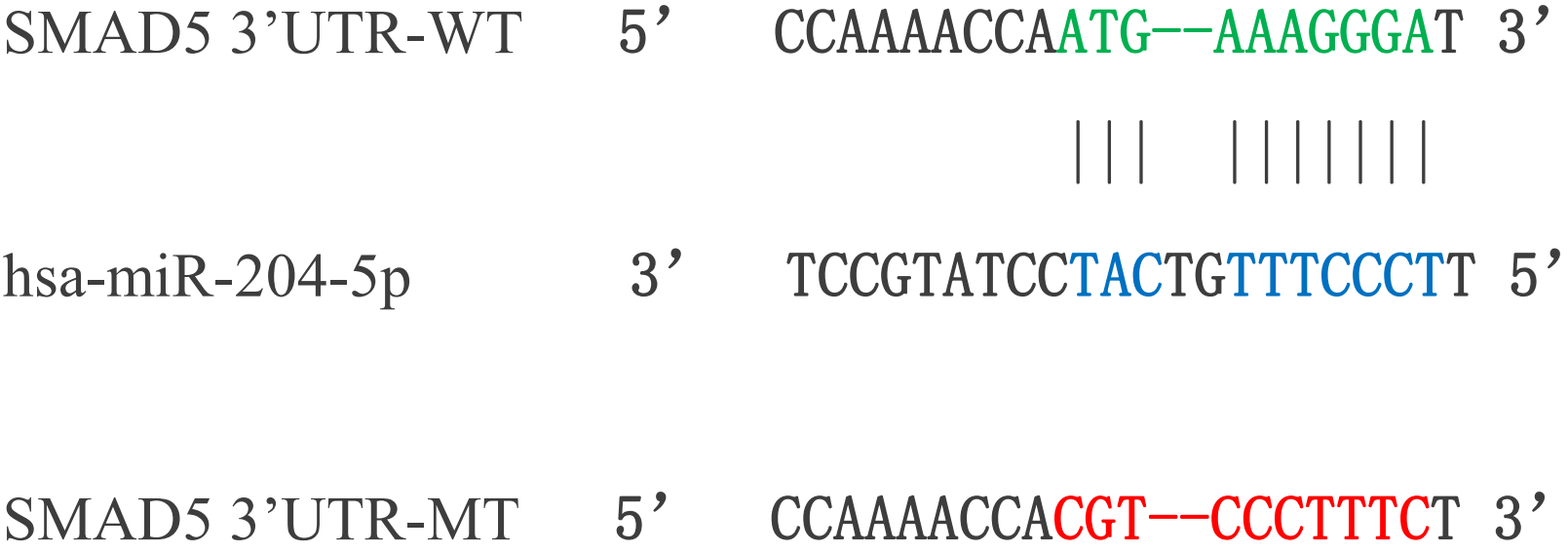

Supplement: Supplementary file 11 [file Table_11.DOCX]

**Raw data of Fig3A(a)**


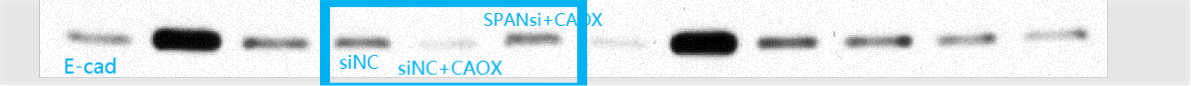

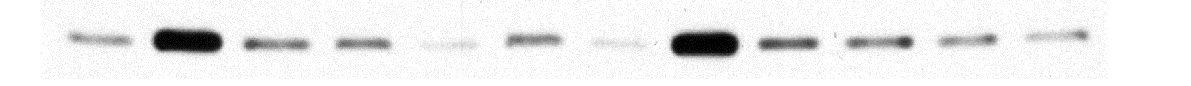

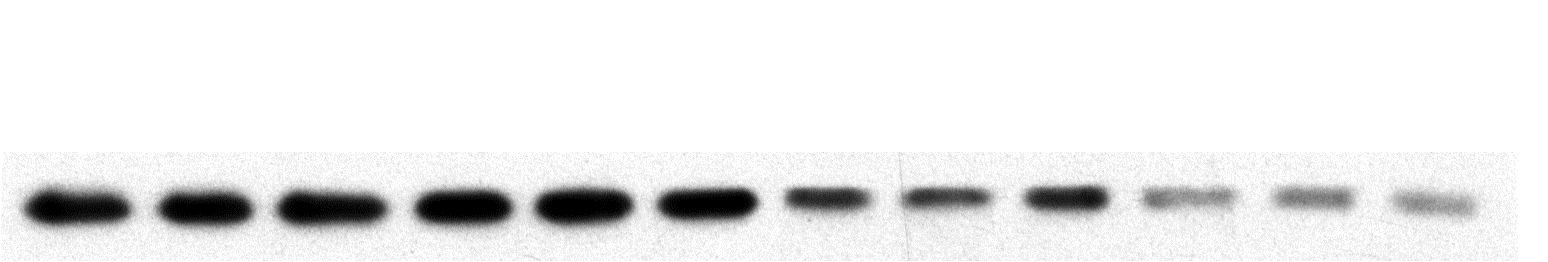

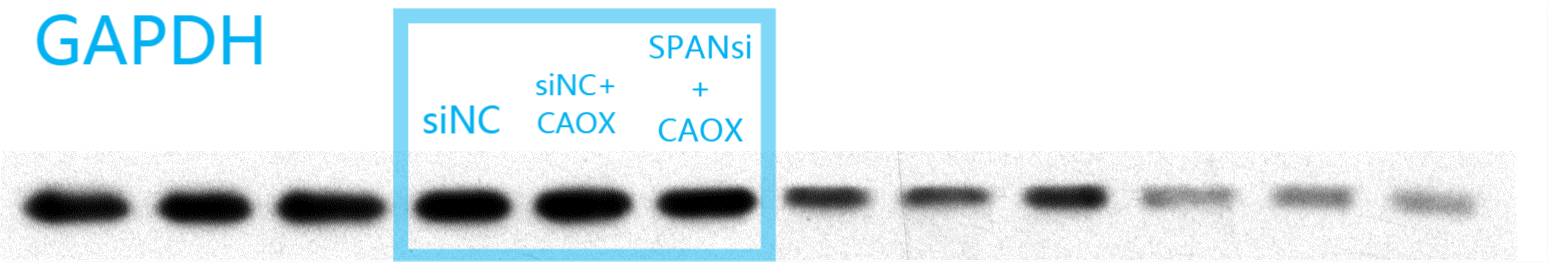

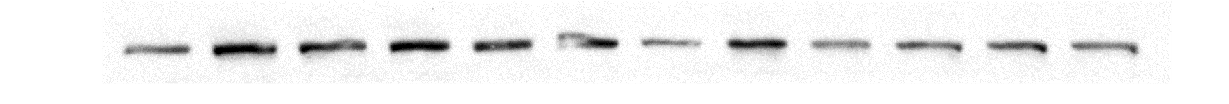

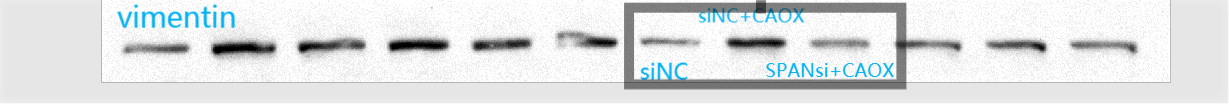

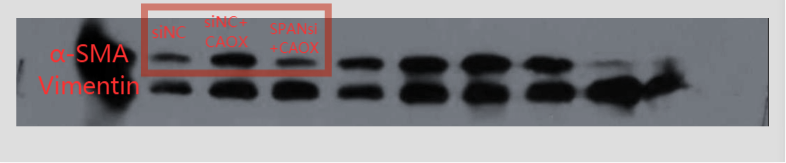

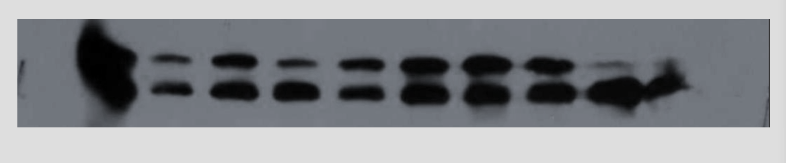


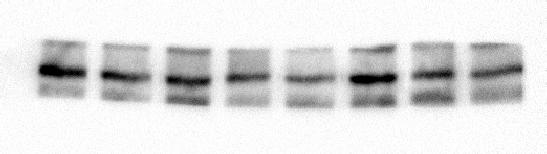


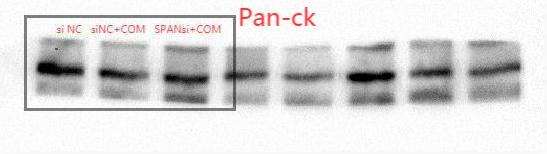


**Raw data of**

**Fig5A(b)**
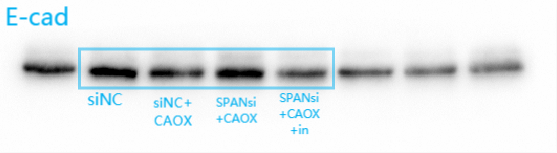

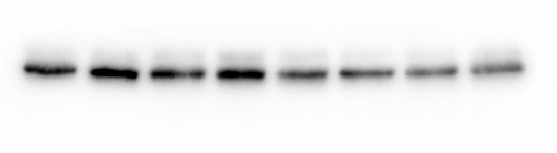

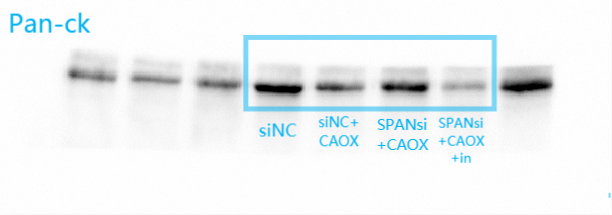

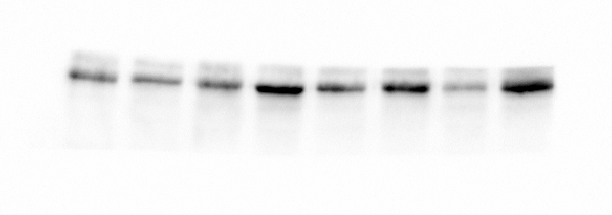

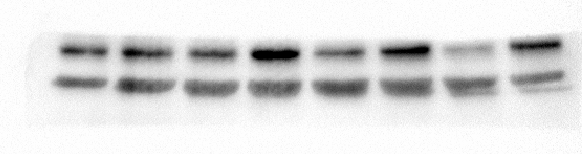

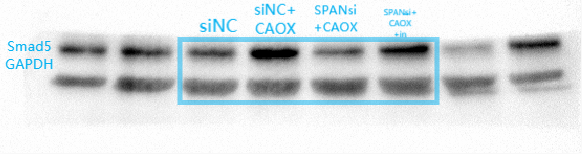

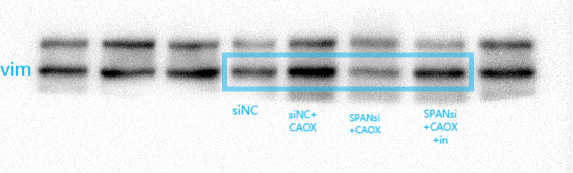

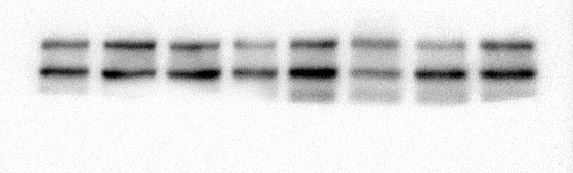

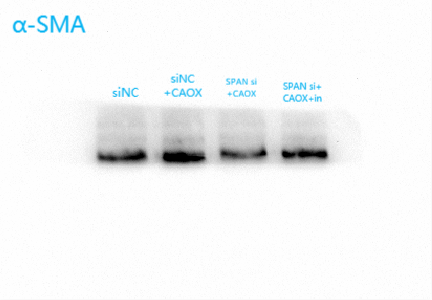

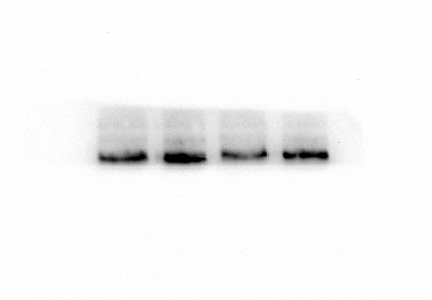


**Raw data of**

**Fig5C(a)**

**
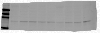

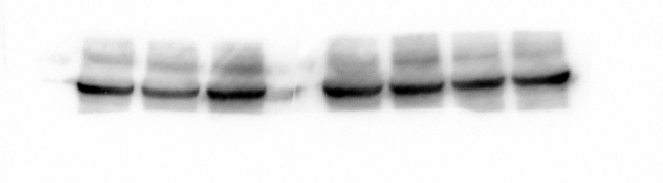

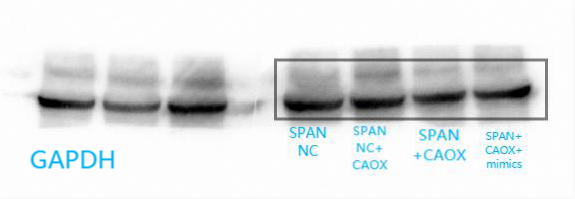

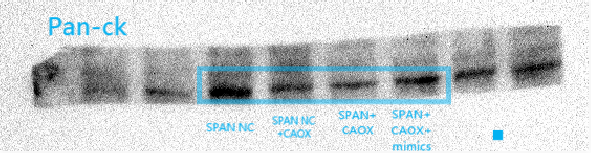

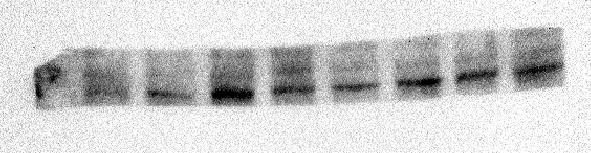

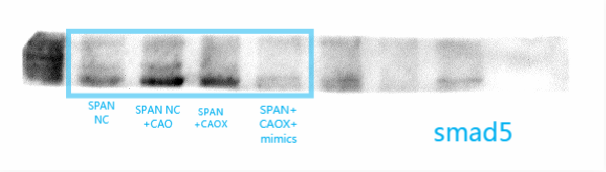

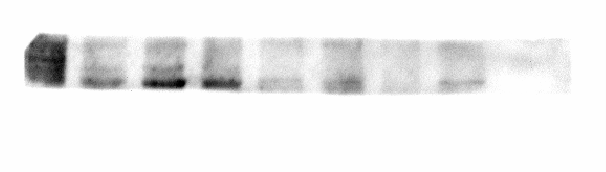

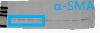
**

Supplement: Supplementary file 13 [file Table_13.DOC]

**Fig1A**


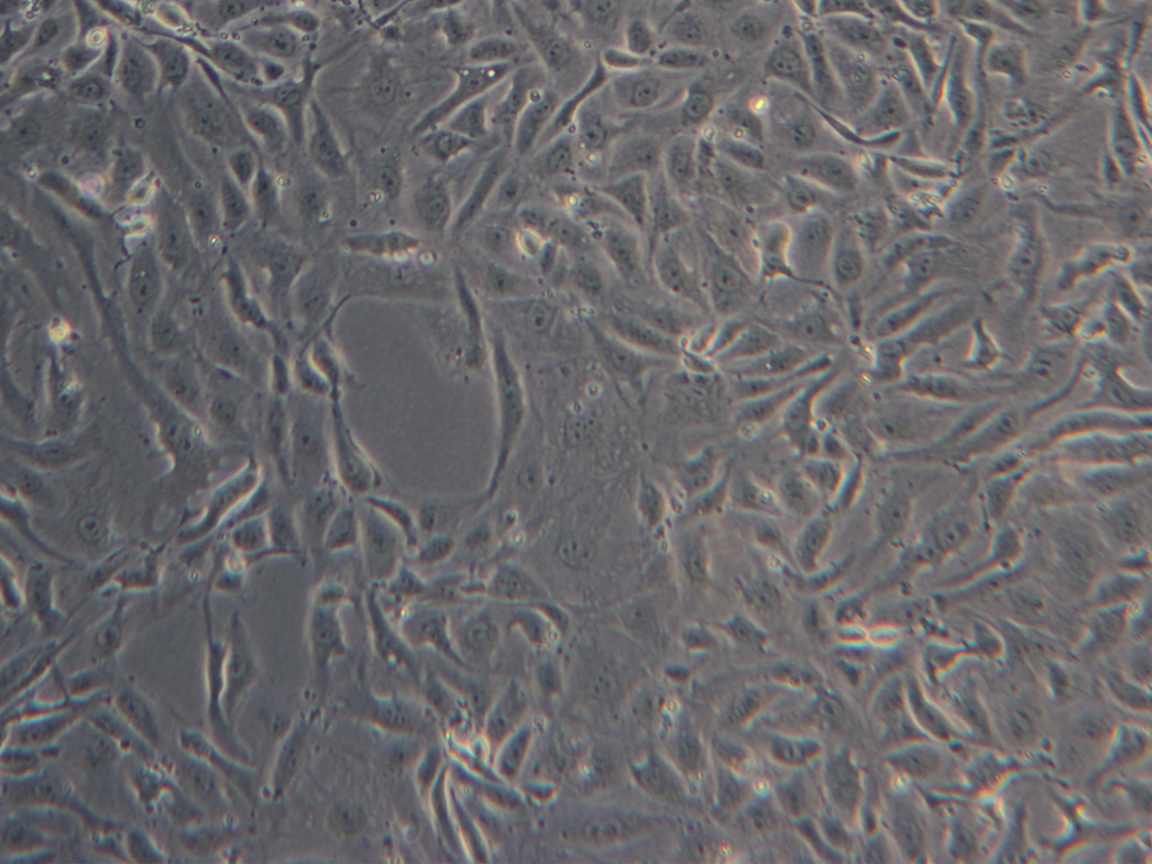


**Fig1B**


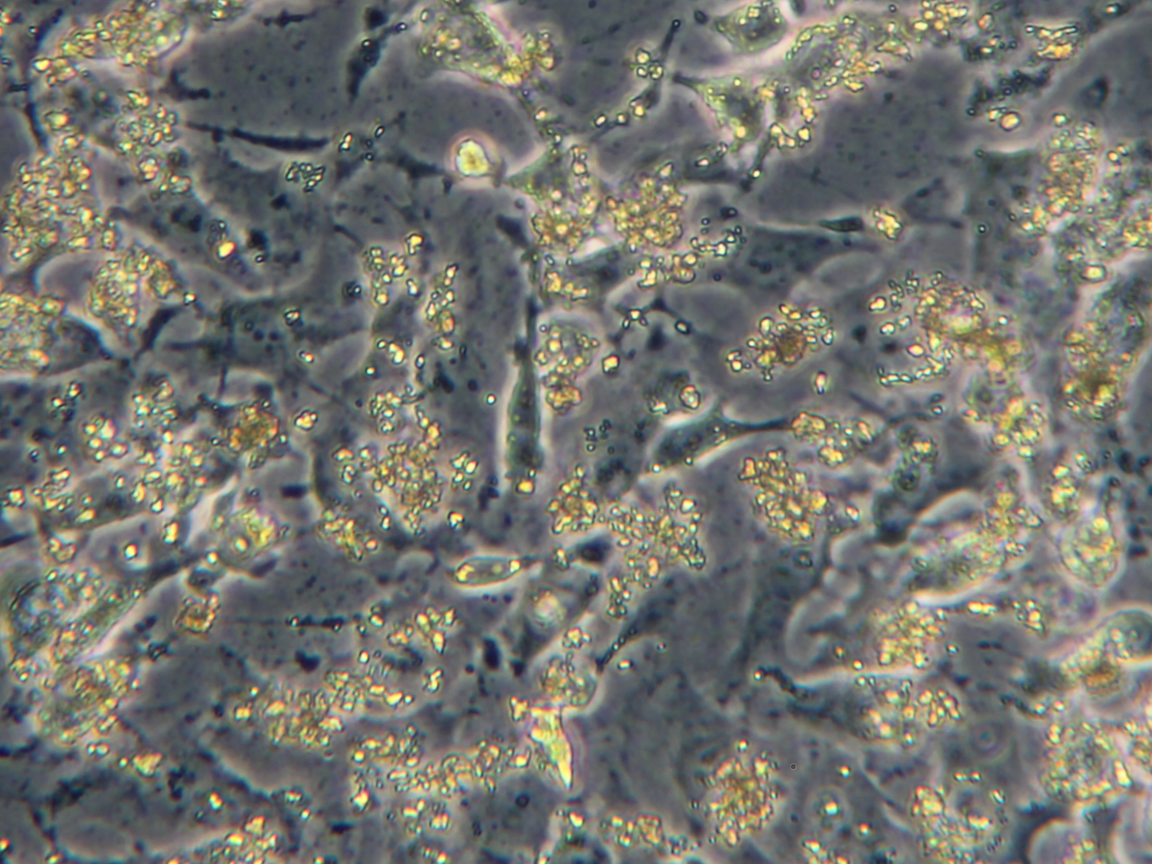

Supplement: Supplementary file 14 [file Table_14.DOC]
